# Machine Learning–Based Prediction of Acute Kidney Injury Following Pediatric Cardiac Surgery: Model Development and Validation Study

**DOI:** 10.2196/41142

**Published:** 2023-01-05

**Authors:** Xiao-Qin Luo, Yi-Xin Kang, Shao-Bin Duan, Ping Yan, Guo-Bao Song, Ning-Ya Zhang, Shi-Kun Yang, Jing-Xin Li, Hui Zhang

**Affiliations:** 1 Department of Nephrology The Second Xiangya Hospital of Central South University Changsha China; 2 Department of Cardiovascular Surgery The Second Xiangya Hospital of Central South University Changsha China; 3 Information Center The Second Xiangya Hospital of Central South University Changsha China; 4 Department of Nephrology The Third Xiangya Hospital of Central South University Changsha China; 5 Department of Cardiovascular Surgery Xiangya Hospital of Central South University Changsha China; 6 Department of Pediatrics Xiangya Hospital of Central South University Changsha China

**Keywords:** cardiac surgery, acute kidney injury, pediatric, machine learning

## Abstract

**Background:**

Cardiac surgery–associated acute kidney injury (CSA-AKI) is a major complication following pediatric cardiac surgery, which is associated with increased morbidity and mortality. The early prediction of CSA-AKI before and immediately after surgery could significantly improve the implementation of preventive and therapeutic strategies during the perioperative periods. However, there is limited clinical information on how to identify pediatric patients at high risk of CSA-AKI.

**Objective:**

The study aims to develop and validate machine learning models to predict the development of CSA-AKI in the pediatric population.

**Methods:**

This retrospective cohort study enrolled patients aged 1 month to 18 years who underwent cardiac surgery with cardiopulmonary bypass at 3 medical centers of Central South University in China. CSA-AKI was defined according to the 2012 Kidney Disease: Improving Global Outcomes criteria. Feature selection was applied separately to 2 data sets: the preoperative data set and the combined preoperative and intraoperative data set. Multiple machine learning algorithms were tested, including K-nearest neighbor, naive Bayes, support vector machines, random forest, extreme gradient boosting (XGBoost), and neural networks. The best performing model was identified in cross-validation by using the area under the receiver operating characteristic curve (AUROC). Model interpretations were generated using the Shapley additive explanations (SHAP) method.

**Results:**

A total of 3278 patients from one of the centers were used for model derivation, while 585 patients from another 2 centers served as the external validation cohort. CSA-AKI occurred in 564 (17.2%) patients in the derivation cohort and 51 (8.7%) patients in the external validation cohort. Among the considered machine learning models, the XGBoost models achieved the best predictive performance in cross-validation. The AUROC of the XGBoost model using only the preoperative variables was 0.890 (95% CI 0.876-0.906) in the derivation cohort and 0.857 (95% CI 0.800-0.903) in the external validation cohort. When the intraoperative variables were included, the AUROC increased to 0.912 (95% CI 0.899-0.924) and 0.889 (95% CI 0.844-0.920) in the 2 cohorts, respectively. The SHAP method revealed that baseline serum creatinine level, perfusion time, body length, operation time, and intraoperative blood loss were the top 5 predictors of CSA-AKI.

**Conclusions:**

The interpretable XGBoost models provide practical tools for the early prediction of CSA-AKI, which are valuable for risk stratification and perioperative management of pediatric patients undergoing cardiac surgery.

## Introduction

An increasing number of pediatric patients worldwide undergo cardiac surgery each year for various reasons, including congenital heart disease and acquired cardiac conditions [1]. Cardiac surgery–associated acute kidney injury (CSA-AKI), characterized by an abrupt decrease in renal function, is a major complication following pediatric cardiac surgery. The reported incidence of CSA-AKI among pediatric patients undergoing cardiac surgery ranges from 5% to 42% [2]. Importantly, CSA-AKI is associated with significantly increased morbidity and mortality, prolonged length of hospital stay, and an increased risk of chronic kidney disease [3-5].

The early prediction of CSA-AKI could significantly improve the implementation of preventive and therapeutic strategies during the perioperative periods. Specifically, preoperative prediction could facilitate surgery risk assessment and prevention of CSA-AKI, and early postoperative prediction could help with the early identification of CSA-AKI for proactive interventions [6]. Therefore, it is of great clinical interest to establish precise prediction models for CSA-AKI to identify high-risk patients and to optimize the perioperative management of pediatric patients undergoing cardiac surgery. Recently, several prediction models combining biomarkers and clinical variables have been established with the goal to predict the development of CSA-AKI in the pediatric population [7-9]. However, those models were limited by small sample sizes, lack of internal and external validation, and additional financial burdens due to the use of novel biomarkers.

The widespread use of machine learning to analyze clinical data derived from electronic health records offers considerable advantages for establishing prediction models. Machine learning is a scientific discipline that uses computer algorithms and learns from data with minimal human intervention [10]. Advanced machine learning algorithms can model nonlinear relationships, analyze complex high-order interactions, and robustly handle multicollinearity among the predictor variables. The application of machine learning has led to significant breakthroughs in various medical fields such as emergency department triage [11], prediction of postinduction hypotension [12], and risk stratification of postcontrast acute kidney injury [13]. Machine learning approaches have also shown promising performance in the early prediction of adult CSA-AKI [14-19], but their predictive performance for CSA-AKI in the pediatric population has not been tested. The primary diseases, underlying pathophysiology, and risk factors of CSA-AKI in pediatric patients are significantly different from those in adult patients [20-22]. Therefore, the existing prediction models for adult CSA-AKI are not applicable to pediatric patients. The objective of this study was to develop and validate machine learning models for predicting the development of CSA-AKI in pediatric patients undergoing cardiac surgery.

## Methods

### Study Design

This study includes patients from 3 distinct medical centers of Central South University in China. The derivation cohort comprised patients admitted at the Second Xiangya Hospital between January 2015 and March 2022. The external validation cohort consisted of patients admitted at Xiangya Hospital between January 2016 and December 2021 and patients admitted at the Third Xiangya Hospital between January 2015 and December 2021. 

### Ethics Approval

This study follows the Declaration of Helsinki and the Transparent Reporting of a multivariable prediction model for Individual Prognosis Or Diagnosis statement [23]. This study protocol was approved by the medical ethics committee of the Second Xiangya Hospital of Central South University (2022-K031). The requirement for informed consent was waived due to the retrospective nature and minimal risk of this study.

### Study Participants

**Figure 1:**
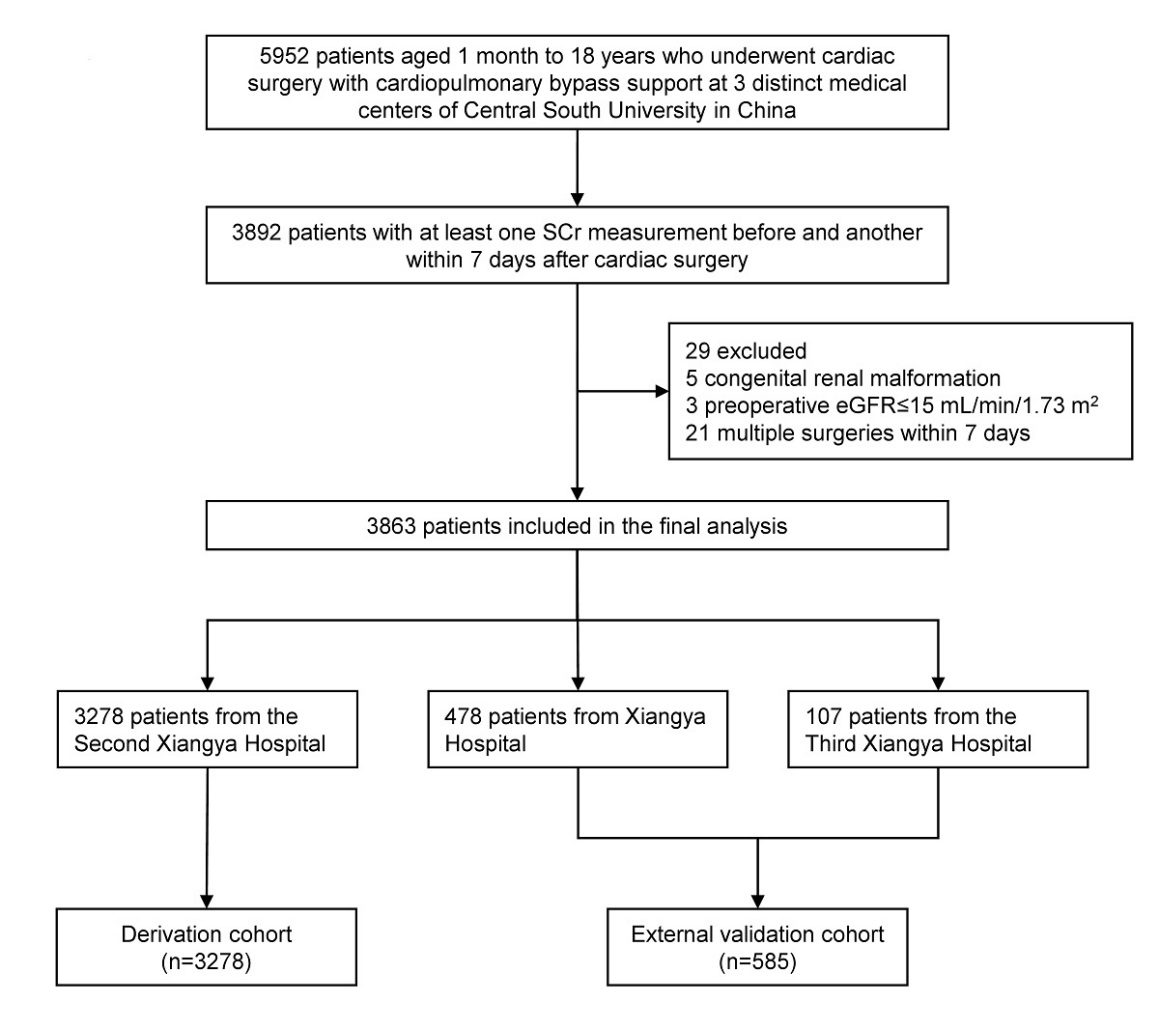
Flow diagram of patient selection. eGFR: estimated glomerular filtration rate; SCr: serum creatinine.

This study includes all pediatric patients aged between 1 month and 18 years who underwent cardiac surgery with cardiopulmonary bypass. We included patients with at least one serum creatinine (SCr) measurement before surgery and another within 7 days after surgery. We excluded patients with congenital renal malformation, preoperative estimated glomerular filtration rate (eGFR) of 15 mL/min/1.73 m^2^ or lower, or multiple surgeries within 7 days. We calculated eGFR using the modified Schwartz equation, or if body length was missing, using the Full Age Spectrum equation [24,25]. Details on patient selection in the derivation and external validation cohorts are provided in Figure 1.

### Predictor Variables

Potential predictors included those considered clinically relevant to the development of CSA-AKI and available in the electronic health records, with less than 30% of such observations missing. Potential predictors were divided into preoperative and intraoperative variables. Preoperative variables included patient demographics, preoperative conditions, laboratory tests, and medications. Preoperative conditions were determined according to the diagnosis on admission, preoperative diagnosis, and preoperative anesthesia interview records. The most recent preoperative measurements were used for laboratory variables. Medications were classified based on the Anatomic Therapeutic Chemical classification system and included if administered within 7 days before the surgery [26]. Intraoperative data were extracted from the records of anesthesia, cardiopulmonary bypass, and surgery. The complexity of the surgeries was determined by the Risk Adjustment for Congenital Heart Surgery-1 (RACHS-1) score, which is a consensus-based tool for short-term mortality risk based on the type of the procedure performed [27]. The volume of blood loss was calculated as the sum of blood loss in operative fields, residual volume in the pump, and any additional loss by other routes. The total fluid balance was calculated as the difference between the total fluid input and total fluid output and corrected for body weight.

### Outcome Measures

The primary outcome was the development of CSA-AKI, which was determined according to the 2012 Kidney Disease: Improving Global Outcomes (KDIGO) clinical practice guideline [28]. CSA-AKI was defined as an increase in SCr level of at least 0.3 mg/dL within 48 hours or 50% within 7 days after surgery compared with the baseline. The most recent SCr value within 90 days prior to the surgery was used as the baseline. Secondary outcomes included CSA-AKI stages 2-3 (defined by the KDIGO criteria), in-hospital mortality, length of stay in the intensive care unit, and length of postoperative hospital stay.

### Statistical Analysis

Descriptive statistics are presented as medians and interquartile ranges for continuous variables and as numbers and percentages for categorical variables. Data distributions were compared using the Mann-Whitney *U* test for continuous variables and chi-square tests for categorical variables. One-hot encoding was performed by preprocessing each categorical variable into binary variables. Missing data were imputed by the random forest method using the missForest package in R [29]. The description of the data types and the missing values for each variable can be found in Table S1 of Multimedia Appendix 1.

### Feature Selection

Predictor variables with near-zero variance, identified as those with the percentages of unique values less than 5%, were removed from the analysis. Subsequently, 4 feature selection methods were used to obtain subsets of the predictor variables for further model development. The methods included Least Absolute Shrinkage and Selection Operator, Boruta algorithm, random forest-recursive feature elimination, and random forest-filtering. The results obtained by the 4 methods were comprehensively evaluated, and the predictor variables that appeared more than 3 times among the 4 methods were ultimately selected to build the model. Feature selection was conducted twice—first to include only the preoperative variables and then by combining the preoperative and intraoperative variables. The glmnet, Boruta, and caret packages in R were used for feature selection.

### Model Development and Validation

For model development, the following machine learning algorithms were applied to the preoperative-only and the combined data sets of the derivation cohort: K-nearest neighbor, naive Bayes, support vector machines, random forest, extreme gradient boosting (XGBoost), and neural networks. We conducted 5 random shuffles of 5-fold cross-validation to ensure an unbiased assessment of model performance and to identify the optimal hyperparameters for each model. Model performance was assessed based on the mean area under the receiver operating characteristic curves (AUROCs) from 5×5 iterations. After that, the best performing machine learning model was chosen for each data set. The caret package in R was used for model development. The details on functions, packages, and tuning parameters used for each machine learning algorithm are provided in Table S2 of Multimedia Appendix 2.

The performance of the final prediction models was further evaluated in the external validation cohort. The metrics for model performance included AUROC, the area under the precision-recall curve (AUPRC), and the calibration plot. AUROC was used as the primary performance metric because it is independent of the thresholds in the setting of class imbalance. AUPRC is known to be more informative for class-imbalanced prediction tasks because it is sensitive to changes in the number of false-positive predictions [30]. In addition, the performance of the final machine learning models was compared with that of the traditional logistic regression models. The framework of model establishment is depicted in Figure S1 of Multimedia Appendix 3.

### Model Interpretations

The Shapley additive explanations (SHAP) method was used to explore the interpretability of the final prediction models. SHAP is a unified approach to the interpretations of model predictions, which provides consistent and locally accurate attribution values for each feature within a prediction model, namely, the SHAP values [31]. Higher SHAP values indicate an increased probability of CSA-AKI. The contribution of the predictor variables to CSA-AKI can be explained as the cumulative effects of variable attributions to the entire output risk for each observation.

### Sensitivity Analysis

Sensitivity analysis was performed to examine the predictive power of the models for CSA-AKI stages 2-3. Model performance was also evaluated in the subgroups, focusing on patients in different age groups (infancy: 1 month to 1 year; childhood: 2-10 years; adolescence: 11-18 years) [32] and patients with different surgical complexities (RACHS-1 score of 2 or lower; RACHS-1 score of 3 or higher). In addition, to translate the prediction models into clinical risk-stratification tools, we identified the low- and high-risk cutoff values of the predictive probabilities. The analyses included sensitivity, specificity, positive and negative predictive values, and positive and negative likelihood ratios. Finally, to evaluate the effect of the imbalanced outcomes on model performance, we applied upsampling or downsampling to generate a balanced derivation cohort for model training. All statistical analyses were performed using R 4.1.2 [33]. A 2-tailed *P* value of <.05 was considered statistically significant.

## Results

### Patient Characteristics

A total of 3863 participants were enrolled in this study, that is, 3278 in the derivation cohort and 585 in the external validation cohort. The baseline characteristics and outcomes of the patients in the derivation and external validation cohorts are shown in Table 1. In the derivation cohort, 564 (17.2%) patients developed CSA-AKI, comprising 356 (10.9%) with stage 1, 142 (4.3%) with stage 2, and 66 (2%) with stage 3. In the external validation cohort, 51 (8.7%) patients developed CSA-AKI, comprising 25 (4.3%) with stage 1, 14 (2.4%) with stage 2, and 12 (2.1%) with stage 3. The comparison of the baseline characteristics and outcomes according to the development of CSA-AKI are provided in Table S3 and Table S4 of Multimedia Appendix 4.

**Table 1:** Baseline characteristics and outcomes of the patients in the derivation and external validation cohorts.

Variables		Derivation cohort (n=3278)	External validation cohort (n=585)
**Demographics**			
	Age (year), median (IQR)	1 (0.5-4)	4 (1-8)
	Sex (male), n (%)	1709 (52.1)	288 (49.2)
	Body length (cm), median (IQR)	79 (65-105)	100 (80-128)
	Weight (kg), median (IQR)	9.5 (6.0-16.0)	15.0 (10.0-22.5)
**ABO blood groups, n (%)**			
	Type A	1100 (33.6)	200 (34.4)
	Type B	744 (22.7)	134 (23)
	Type O	1182 (36.1)	197 (33.8)
	Type AB	252 (7.7)	51 (8.8)
**Preoperative conditions**			
	Cyanotic heart disease, n (%)	740 (22.6)	113 (19.3)
	Pulmonary hypertension, n (%)	1763 (53.8)	241 (41.2)
	Pulmonary infection, n (%)	293 (8.9)	30 (5.1)
	Infective endocarditis, n (%)	44 (1.3)	9 (1.5)
	Previous cardiac surgery, n (%)	181 (5.5)	21 (3.6)
	Genetic disease, n (%)	72 (2.2)	12 (2.1)
	Noncardiac malformation, n (%)	111 (3.4)	15 (2.6)
	Preoperative intensive care, n (%)	168 (5.1)	10 (1.7)
	Preoperative length of stay (day), median (IQR)	4 (2-7)	6 (3-7)
**American Society of Anesthesiologists** **physical status, n (%)**			
	Ⅰ	21 (0.6)	0 (0)
	Ⅱ	589 (18)	120 (21.2)
	Ⅲ	1978 (60.6)	339 (59.8)
	Ⅳ	665 (20.4)	108 (19)
	V	12 (0.4)	0 (0)
**Laboratory tests**			
	Baseline creatinine (µmol/L), median (IQR)	25.3 (20.4-33.5)	44.0 (36.0-53.0)
	Baseline estimated glomerular filtration rate (mL/min/1.73 m^2^), median (IQR)	118.0 (99.5-138.0)	85.0 (72.4-97.1)
	Left ventricular ejection fraction (%), median (IQR)	69 (66-73)	66 (62-70)
	Hemoglobin (g/L), median (IQR)	119 (107-129)	125 (117-135)
	Red blood cell distribution width (%), median (IQR)	13.4 (12.7-14.7)	13.6 (12.9-14.7)
	White blood cells (×10^9^/L), median (IQR)	8.0 (6.5-9.8)	7.6 (6.3-9.5)
	Platelets (×10^9^/L), median (IQR)	314 (253-383)	271 (223-326)
	Dipstick albuminuria, n (%)	57 (2.1)	4 (0.8)
	Blood urea nitrogen (mmol/L), median (IQR)	4.08 (2.96-5.12)	4.16 (3.22-5.05)
	Total bilirubin (µmol/L), median (IQR)	7.4 (5.1-10.9)	7.3 (5.1-11.0)
	Alanine aminotransferase (U/L), median (IQR)	16.4 (11.7-25.3)	14.2 (11.1-19.0)
	Aspartate aminotransferase (U/L), median (IQR)	34.9 (27.4-45.4)	31.4 (25.6-39.1)
	Albumin (g/L), median (IQR)	40.3 (38.2-42.3)	43.1 (41.0-45.4)
	Potassium (mmol/L), median (IQR)	4.80 (4.46-5.12)	4.45 (4.19-4.73)
	Sodium (mmol/L), median (IQR)	138.3 (137.0-139.7)	140.0 (138.7-141.1)
	Chloride (mmol/L), median (IQR)	103.2 (101.6-104.7)	104.1 (102.7-105.5)
	Calcium (mmol/L), median (IQR)	2.40 (2.32-2.49)	2.46 (2.37-2.54)
**Preoperative medications, n (%)**			
	Iodinated contrast media	411 (12.5)	73 (12.5)
	Digoxin	104 (3.2)	11 (1.9)
	Diuretics	316 (9.6)	38 (6.5)
	Nonsteroidal anti-inflammatory drugs	54 (1.6)	12 (2.1)
	Angiotensin converting enzyme inhibitor/angiotensin Ⅱ receptor blocker	61 (1.9)	4 (0.7)
	Nephrotoxic antibiotics	61 (1.9)	1 (0.2)
	Antiviral drugs	163 (5)	1 (0.2)
**Intraoperative variables**			
	Emergent surgery, n (%)	168 (5.1)	14 (2.4)
	Operation time (min), median (IQR)	155 (129-197)	190 (165-230)
	Perfusion time (min), median (IQR)	58 (44-84)	63 (46-90)
	Cross clamp time (min), median (IQR)	33 (23-51)	37 (23-55)
	Cardioversion, n (%)	279 (8.5)	73 (12.5)
	Lowest mean arterial pressure (mmHg), median (IQR)	35 (31-40)	38 (31-45)
	Lowest core temperature (°C), median (IQR)	33.3 (31.8-34.4)	33.3 (31.7-34.7)
	Intraoperative blood loss (mL/kg), median (IQR)	21.4 (14.8-31.3)	20.0 (15.4-26.2)
	Intraoperative fluid balance (%), median (IQR)	–0.7 (–1.9 to 0.1)	1.6 (–0.2 to 2.9)
**Risk Adjustment for Congenital Heart Surgery-1 score, n (%)**			
	1	480 (14.9)	92 (16.2)
	2	2032 (63)	386 (67.8)
	3	653 (20.3)	84 (14.8)
	4	59 (1.8)	7 (1.2)
**Outcomes**			
	Acute kidney injury, n (%)	564 (17.2)	51 (8.7)
	Acute kidney injury stages 2-3, n (%)	208 (6.3)	26 (4.4)
	In-hospital mortality, n (%)	38 (1.2)	5 (0.9)
	Intensive care unit length of stay (day), median (IQR)	2 (1-3)	1 (1-2)
	Hospital length of stay (day), median (IQR)	8 (7-13)	8 (7-9)

### Predictor Variables

A total of 25 preoperative variables were selected as predictors of CSA-AKI by the 4 feature selection methods and included in the machine learning models (Table S5 of Multimedia Appendix 5). When preoperative and intraoperative variables were combined, a total of 27 variables were incorporated; of those, 20 were preoperative variables and 7 were intraoperative variables (Table S6 of Multimedia Appendix 5).

### Model Performance

**Figure 2:**
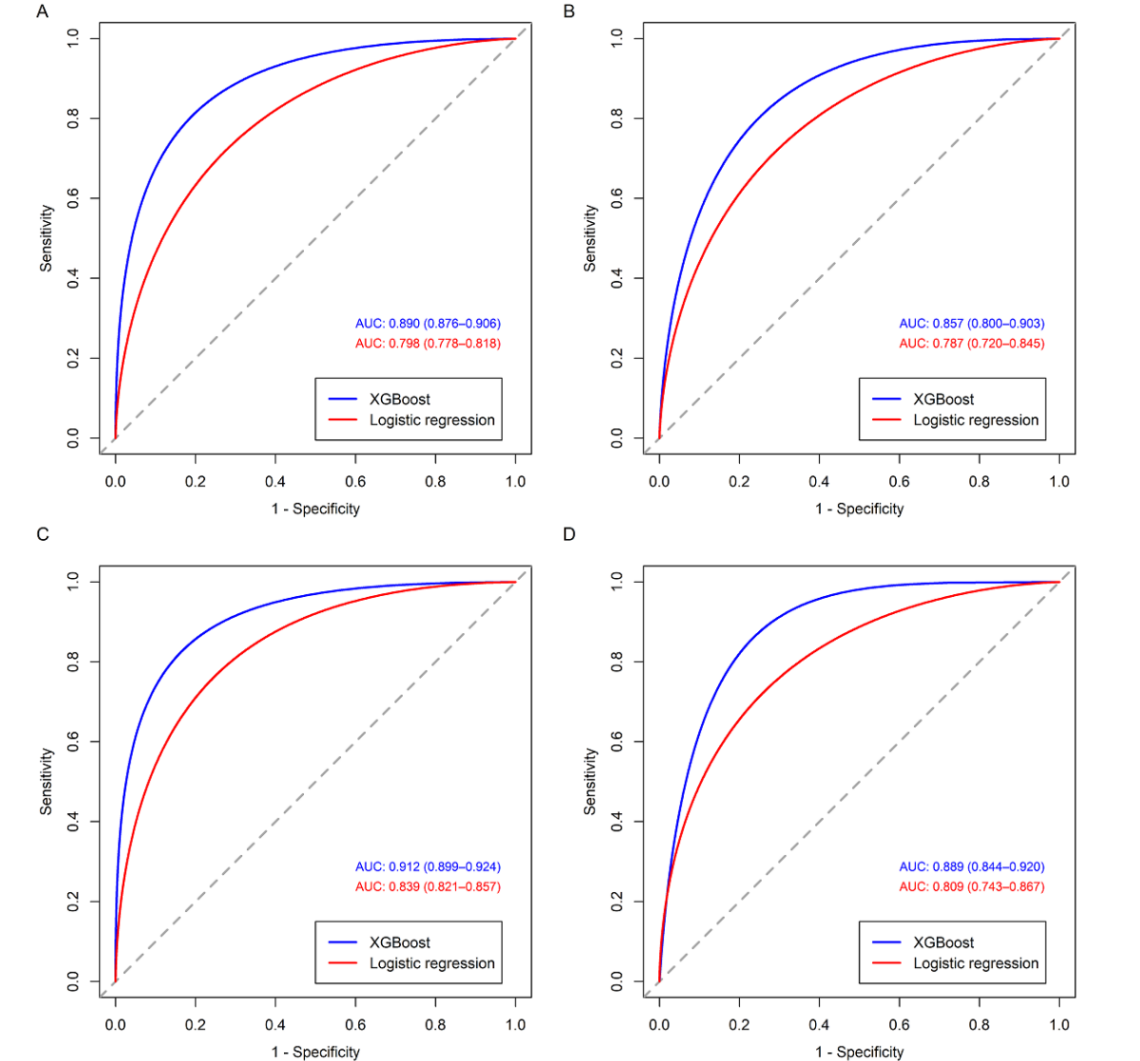
Receiver operating characteristic curves of the extreme gradient boosting and traditional logistic regression models. (A-B) Receiver operating characteristic curves of the models with only the preoperative variables in the (A) derivation and (B) external validation cohorts. (C-D) Receiver operating characteristic curves of the models with the preoperative and intraoperative variables in the (C) derivation and (D) external validation cohorts. AUC: area under the curve; XGBoost: extreme gradient boosting.

Among the considered machine learning models, the XGBoost model achieved the best performance on both the preoperative-only and the combined data sets, with a mean AUROC of 0.795 and 0.832 in cross-validation, respectively (Table S7 of Multimedia Appendix 6). The inclusion of the intraoperative variables improved the predictive power of all considered machine learning models. The AUROCs of the XGBoost and traditional logistic regression models are shown in Figure 2. The XGBoost model with only the preoperative variables exhibited an AUROC of 0.890 (95% CI 0.876-0.906) in the derivation cohort and 0.857 (95% CI 0.800-0.903) in the external validation cohort. When the intraoperative variables were included, the AUROC of the XGBoost model increased to 0.912 (95% CI 0.899-0.924) in the derivation cohort and 0.889 (95% CI 0.844-0.920) in the external validation cohort. The XGBoost algorithm achieved higher AUROCs than traditional logistic regression for both the derivation and external validation cohorts. Details on other performance metrics, including the AUPRCs and the calibration plots, are provided in Figures S2 and S3 of Multimedia Appendix 7. Due to the imbalance in the proportions of patients with and without CSA-AKI, the AUPRC of the XGBoost model using the combined data set was 0.747 in the derivation cohort and 0.476 in the external validation cohort. The Brier scores of the XGBoost model for the combined data set were 0.085 and 0.060 in the derivation and the external validation cohorts, respectively. The final XGBoost models can be accessed at LuoXiaoqin123/pediatric-CSA-AKI (GitHub) [34].

### Model Interpretations

**Figure 3:**
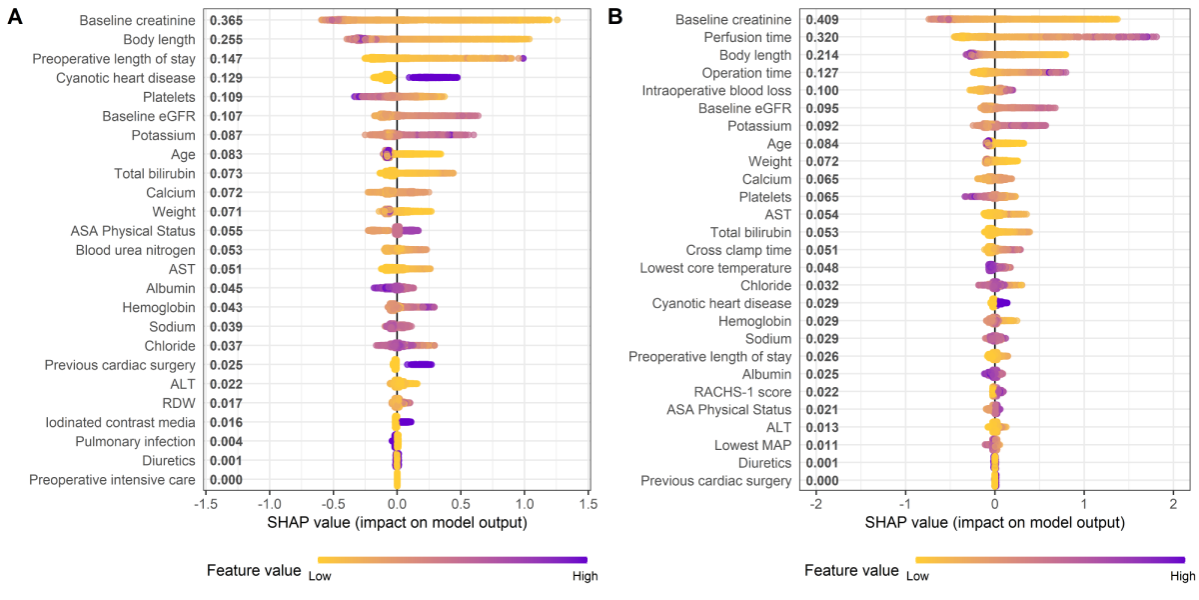
Shapley additive explanations summary plots of the extreme gradient boosting models for cardiac surgery–associated acute kidney injury. (A) Shapley additive explanations summary plot of the extreme gradient boosting model with only the preoperative variables. (B) Shapley additive explanations summary plot of the extreme gradient boosting model with the preoperative and intraoperative variables. A dot is created for each feature attribution in calculating the output risk for each observation. ALT: alanine aminotransferase; ASA: American Society of Anesthesiologists; AST: aspartate aminotransferase; eGFR: estimated glomerular filtration rate; MAP: mean arterial pressure; RACHS: Risk Adjustment for Congenital Heart Surgery; RDW: red blood cell distribution width; SHAP: Shapley additive explanations.

The SHAP summary plots of the XGBoost models are shown in Figure 3, which illustrates how high and low values of each feature relate to SHAP values. The plots also identify the features that influenced the model predictions the most. Baseline SCr, perfusion time, body length, operation time, and intraoperative blood loss were the top 5 predictor variables associated with CSA-AKI in the XGBoost model using the combined data set. The SHAP dependence plots of the XGBoost model with the combined data set are shown in Figures S4 of Multimedia Appendix 8, which shows the nonlinear association between the predictors and the risk of CSA-AKI. The plots show how changes in a single feature can affect model output.

### Sensitivity Analysis

The XGBoost models showed good predictive performance for CSA-AKI stages 2-3, with AUROCs higher than 0.85 in both the derivation and the external validation cohorts, respectively (Figure S5 of Multimedia Appendix 9). When the models were applied to patients in different age groups or patients with different surgical complexities, the performance of the models remained stable (Figures S6-S10 of Multimedia Appendix 10). The low- and high-risk cutoff values were identified to facilitate clinical applications of the XGBoost models. The diagnostic test characteristics of the models at the low- and high-risk cutoff points are shown in Table 2. For example, the XGBoost model applied to the combined data set yields the following clinically relevant threshold values. The low-risk cutoff value is 0.099 with sensitivity of 95% and a negative predictive value of 98.3%; this value captures the vast majority of patients with CSA-AKI and leaves out only a small proportion of those that are falsely negative. The high-risk cutoff value of 0.374 has specificity of 95% and a positive likelihood ratio of 12.12. Therefore, the low-risk cutoff is appropriate for low-level interventions, while the high-risk cutoff value can be used to identify patients at high risk of CSA-AKI who require more intensive interventions. Additionally, when we used the balanced derivation cohort by upsampling or downsampling to train the models, the models still achieved promising predictive performance (Figures S11 and S12 of Multimedia Appendix 11).

**Table 2:** Diagnostic test characteristics of the extreme gradient boosting models at the low- and high-risk cutoff points.

Models, cohorts				Cutoff value		Sensitivity (%)		Specificity (%)		Positive predictive value (%)		Negative predictive value (%)		Positive likelihood ratio		Negative likelihood ratio
**Models with only preoperative variables**																
	**Derivation cohort**															
		Low-risk cutoff	0.103		95		52.7		29.4		98.1		2.01		0.09	
		High-risk cutoff	0.365		55.3		95		69.8		91.1		11.12		0.47	
	**External validation cohort**															
		Low-risk cutoff	0.103		84.3		80.1		28.9		98.2		4.25		0.20	
		High-risk cutoff	0.365		7.8		99.8		80		91.9		41.88		0.92	
**Models with preoperative and intraoperative variables**																
	**Derivation cohort**															
		Low-risk cutoff	0.099		95		58.3		32.2		98.3		2.28		0.09	
		High-risk cutoff	0.374		60.3		95		71.6		92		12.12		0.42	
	**External validation cohort**															
		Low-risk cutoff	0.099		80.4		80.5		28.3		97.7		4.13		0.24	
		High-risk cutoff	0.374		27.5		98.7		66.7		93.4		20.94		0.74	

## Discussion

### Principal Findings

In this multicenter retrospective study, we developed and externally validated prediction models for pediatric CSA-AKI by using machine learning approaches. Multiple machine learning algorithms were tested in the process of model development, with the XGBoost algorithm ultimately identified as offering the strongest discrimination. In addition, the XGBoost models showed promising predictive performance on both the preoperative-only and combined data sets, demonstrating their potential usefulness for predicting pediatric CSA-AKI. To the best of our knowledge, our study is the first to establish machine learning models for CSA-AKI in the pediatric population that are valuable for risk stratification and clinical decision-making.

Previous studies have shown the advantages of machine learning algorithms in predicting CSA-AKI in adults [14-19]. Lee et al [14] were the first to apply machine learning approaches to predict all stages of CSA-AKI in adults. Their study showed that XGBoost performed better in predicting CSA-AKI than either the traditional logistic regression or risk scores. Additional prediction models based on machine learning algorithms were developed to predict CSA-AKI in the Chinese adult population [18]. Another study established the machine learning models that incorporated the intraoperative time-series and other features to predict adult CSA-AKI. In that study, the ensemble model (random forest + XGBoost) showed the best predictive performance [19]. However, the primary disease, comorbid conditions, and renal physiology in the pediatric population differ significantly from those in adults, which makes the adult prediction models unsuitable for predicting CSA-AKI in pediatric patients [35]. To date, no prediction model for CSA-AKI with prospective applications of machine learning techniques has been established for infants and children. In this study, the models using the XGBoost algorithm had the strongest predictive power among the considered machine learning models. The XGBoost models were further validated using an external validation cohort, and they exhibited consistent predictive performance for pediatric CSA-AKI. Additionally, the sensitivity analyses showed that the XGBoost models displayed comparable predictive performance in most subsets of patients grouped by age or complexity of surgery. Overall, the results demonstrated the robustness and applicability of the XGBoost models in pediatric patients undergoing cardiac surgery.

Both preoperative and intraoperative factors proved to contribute to the prediction of postoperative AKI. Tseng et al [19] emphasized the value of intraoperative features that reflected rapid physiologic changes during surgery relevant to the prediction of CSA-AKI in adults. Another retrospective study found that, by integrating preoperative and intraoperative features, the model for postoperative AKI could reclassify 40% of the false-negative patients from the preoperative model [36]. Our study shows that the XGBoost model combining the preoperative and intraoperative variables achieved better predictive performance than that with the preoperative variables only. Nevertheless, both models exhibited adequate predictive power and potential utility for the preoperative or early postoperative prediction of pediatric CSA-AKI. The preoperative prediction could assist practitioners in evaluating the risk of surgery and support the implementation of preventive strategies. Early postoperative prediction is useful for optimizing postoperative management and care plans, such as continuous assessment of renal function, hemodynamic monitoring, avoidance of nephrotoxin, or renal replacement therapy.

The SHAP method was used to uncover the black box of the XGBoost models. This method is a model-agnostic explanation technique that has been widely used to interpret the contribution of predictors to the model output [37,38]. Consistent with an updated systemic review, we found that lower baseline SCr, longer perfusion time, longer operation time, higher baseline eGFR, younger age, and lower body weight are associated with the development of pediatric CSA-AKI [3,39]. We also identified body length, intraoperative blood loss, serum potassium, and serum calcium as important predictors of CSA-AKI. Notably, 3 of the top 5 important predictors were intraoperative variables, suggesting that the surgical procedure itself has a significant impact on the occurrence of CSA-AKI. During cardiac surgery, significant pathophysiological changes may occur, such as ischemia-reperfusion injury, inflammation response, or activation of coagulation pathways, which may lead to renal injury and dysfunction [40]. Additionally, the conventional logistic regression used in previous studies is limited to revealing the linear relationship between the predictors and CSA-AKI [41-43]. In contrast, the SHAP dependence plots in our study reflected complex nonlinear relationships, which can assist in understanding the association between the changes in predictor variables and the risk of CSA-AKI.

Our findings have significant clinical implications. First, the low- and high-risk cutoff values were identified to promote the clinical application of the XGBoost models. This should allow the care team to identify the patients at high risk of CSA-AKI and to develop optimal perioperative management strategies. Second, our models used the preoperative and intraoperative variables that are routinely collected in clinical practice, thus adding no extra laboratory tests or financial burdens to the standard clinical care procedures. Third, the discovery of modifiable predictors may promote early interventions to mitigate the risk of CSA-AKI.

### Limitations

Our study has several limitations. First, data were retrospectively collected from electronic health records. Second, the study population was restricted to tertiary medical institutions, as pediatric cardiac surgery is typically not offered in primary health care institutions in China. Thus, the applicability of our prediction models needs further validation in diverse populations. Third, the urine output criteria were not used to define CSA-AKI because hourly urine output data were not available for most patients. However, given the routine use of diuretics in the intraoperative and postoperative periods to maintain urine output, few patients with CSA-AKI were missed in this study. Finally, the causality between the predictors and CSA-AKI needs further exploration. Randomized controlled trials would need to be performed to verify whether the modification of certain predictors can prevent the occurrence of CSA-AKI.

### Conclusion

Our study demonstrates the applicability of machine learning approaches in predicting the development of CSA-AKI in the pediatric population. The XGBoost models had consistent and clinically applicable performance in the derivation and external validation cohorts, which indicated their robustness and expandability. Additionally, the predictive value of the preoperative and intraoperative factors was demonstrated by the improved performance of the model when these factors were combined. Ultimately, our models should prove useful in assisting practitioners with risk stratification and clinical decision-making in pediatric patients undergoing cardiac surgery.

## Appendix

Multimedia Appendix 1 Data types and missing values for the variables of interest.

Multimedia Appendix 2 Functions, packages, and tuning parameters used for each machine learning algorithm.

Multimedia Appendix 3 Illustration of the framework of model establishment.

Multimedia Appendix 4 Baseline characteristics and outcomes of patients with and without cardiac surgery–associated acute kidney injury in the derivation and external validation cohorts.

Multimedia Appendix 5 Predictor variables selected by the 4 feature selection methods.

Multimedia Appendix 6 Performance of the machine learning models in cross-validation.

Multimedia Appendix 7 Performance of the extreme gradient boosting models for cardiac surgery–associated acute kidney injury.

Multimedia Appendix 8 Shapley additive explanations dependence plots for the association between the predictors and cardiac surgery–associated acute kidney injury.

Multimedia Appendix 9 Receiver operating characteristic curves of the extreme gradient boosting models for cardiac surgery–associated acute kidney injury stages 2-3.

Multimedia Appendix 10 Receiver operating characteristic curves of the extreme gradient boosting models for cardiac surgery–associated acute kidney injury in the subgroups.

Multimedia Appendix 11 Receiver operating characteristic curves of the extreme gradient boosting models for cardiac surgery–associated acute kidney injury trained on the balanced derivation cohort.

## Abbreviations

AUPRC: area under the precision-recall curve
AUROC: area under the receiver operating characteristic curve
CSA-AKI: cardiac surgery–associated acute kidney injury
eGFR: estimated glomerular filtration rate
KDIGO: Kidney Disease: Improving Global Outcomes
RACHS-1: Risk Adjustment for Congenital Heart Surgery-1
SCr: serum creatinine
SHAP: Shapley additive explanations
XGBoost: extreme gradient boosting
