# Supplementary material for: Machine Learning–Based Prediction of Acute Kidney Injury Following Pediatric Cardiac Surgery: Model Development and Validation Study
Source: J Med Internet Res. 2023 Jan 5;25:e41142. doi: 10.2196/41142 (PMC9893730; doi:10.2196/41142)
Supplement: Multimedia Appendix 1 [file jmir_v25i1e41142_app1.pdf]

Table S1. Data types and missing values for the variables of interest.

Variables	Data types	Missing values, n (%)	
		Derivation cohort	External validation cohort
Age	Continuous	0 (0)	0 (0)
Sex	Categorical	0 (0)	0 (0)
Body length	Continuous	115 (3.5)	98 (16.8)
Weight	Continuous	0 (0)	1 (0.2)
ABO blood groups	Categorical	0 (0)	3 (0.5)
Cyanotic heart disease	Categorical	0 (0)	0 (0)
Pulmonary hypertension	Categorical	0 (0)	0 (0)
Pulmonary infection	Categorical	0 (0)	0 (0)
Infective endocarditis	Categorical	0 (0)	0 (0)
Previous cardiac surgery	Categorical	0 (0)	0 (0)
Genetic disease	Categorical	0 (0)	0 (0)
Noncardiac malformation	Categorical	0 (0)	0 (0)
Preoperative intensive care	Categorical	0 (0)	0 (0)
Preoperative length of stay	Continuous	0 (0)	0 (0)
American Society of Anesthesiologists physical status	Continuous	13 (0.4)	18 (3.1)
Baseline creatinine	Continuous	0 (0)	0 (0)
Baseline estimated glomerular filtration rate	Continuous	0 (0)	0 (0)
Left ventricular ejection fraction	Continuous	57 (1.7)	54 (9.2)
Hemoglobin	Continuous	6 (0.2)	4 (0.7)
Red blood cell distribution width	Continuous	7 (0.2)	4 (0.7)
White blood cells	Continuous	6 (0.2)	4 (0.7)
Platelets	Continuous	6 (0.2)	4 (0.7)
Dipstick albuminuria	Categorical	542 (16.5)	60 (10.3)
Blood urea nitrogen	Continuous	1 (0.03)	0 (0)
Total bilirubin	Continuous	3 (0.09)	1 (0.2)
Alanine aminotransferase	Continuous	3 (0.09)	1 (0.2)
Aspartate aminotransferase	Continuous	4 (0.1)	1 (0.2)
Albumin	Continuous	2 (0.06)	1 (0.2)
Potassium	Continuous	28 (0.9)	5 (0.9)
Sodium	Continuous	28 (0.9)	5 (0.9)
Chloride	Continuous	28 (0.9)	5 (0.9)
Calcium	Continuous	28 (0.9)	5 (0.9)
Iodinated contrast media	Categorical	0 (0)	0 (0)

Digoxin	Categorical	0 (0)	0 (0)
Diuretics	Categorical	0 (0)	0 (0)
Nonsteroidal anti-inflammatory drugs	Categorical	0 (0)	0 (0)
Angiotensin converting enzyme inhibitor/angiotensin II receptor blocker	Categorical	0 (0)	0 (0)
Nephrotoxic antibiotics	Categorical	0 (0)	0 (0)
Antiviral drugs	Categorical	0 (0)	0 (0)
Emergent surgery	Categorical	0 (0)	0 (0)
Operation time	Continuous	11 (0.3)	18 (3.1)
Perfusion time	Continuous	11 (0.3)	0 (0)
Cross clamp time	Continuous	11 (0.3)	0 (0)
Cardioversion	Categorical	11 (0.3)	0 (0)
Lowest mean arterial pressure	Continuous	14 (0.4)	1 (0.2)
Lowest core temperature	Continuous	23 (0.7)	2 (0.3)
Intraoperative blood loss	Continuous	13 (0.4)	19 (3.2)
Intraoperative fluid balance	Continuous	13 (0.4)	19 (3.2)
Risk Adjustment for Congenital Heart Surgery 1 score	Continuous	54 (1.6)	16 (2.7)
