# Supplementary material for: Machine Learning–Based Prediction of Acute Kidney Injury Following Pediatric Cardiac Surgery: Model Development and Validation Study
Source: J Med Internet Res. 2023 Jan 5;25:e41142. doi: 10.2196/41142 (PMC9893730; doi:10.2196/41142)
Supplement: Multimedia Appendix 2 [file jmir_v25i1e41142_app2.pdf]

Table S2. Functions, packages, and tuning parameters used for each machine learning algorithm.

Algorithms	Functions	Packages	Tuning parameters
K-nearest neighbor	knn	base	k
Naïve Bayes	naive_bayes	naivebayes	Laplace correction, distribution type, bandwidth adjustment
Support vector machines	svmRadial	kernlab	Sigma, cost
Random forest	rf	randomForest	Number of randomly selected predictors
Extreme gradient boosting	xgbTree	xgboost	Boosting iterations, max tree depth, shrinkage, minimum loss reduction, subsample ratio of columns, minimum sum of instance weight, subsample percentage
Neural network	nnet	nnet	Number of hidden units, weight decay
