# Supplementary figures and images for: Machine Learning–Based Prediction of Acute Kidney Injury Following Pediatric Cardiac Surgery: Model Development and Validation Study
Source: J Med Internet Res. 2023 Jan 5;25:e41142. doi: 10.2196/41142 (PMC9893730; doi:10.2196/41142)

**Figure S1.** Illustration to the framework of model establishment.

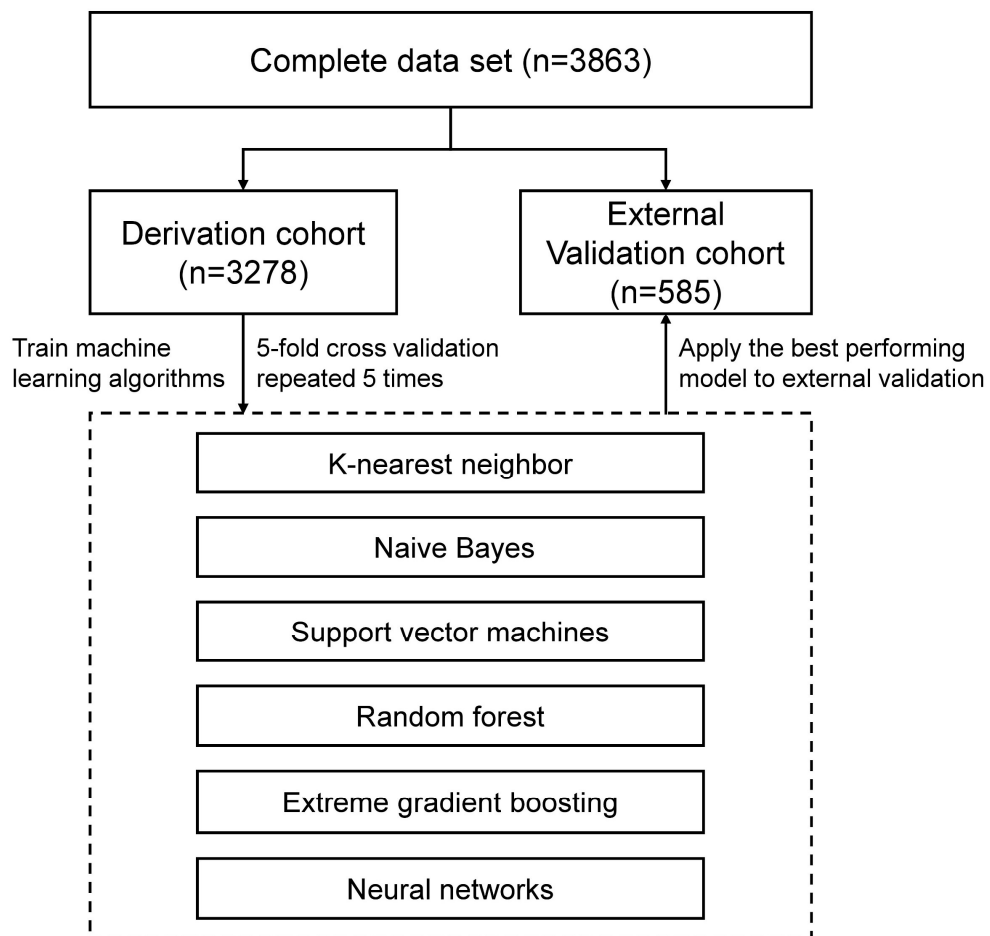

Supplement: Multimedia Appendix 3 [file jmir_v25i1e41142_app3.pdf]
