# Supplementary material for: Machine Learning–Based Prediction of Acute Kidney Injury Following Pediatric Cardiac Surgery: Model Development and Validation Study
Source: J Med Internet Res. 2023 Jan 5;25:e41142. doi: 10.2196/41142 (PMC9893730; doi:10.2196/41142)
Supplement: Multimedia Appendix 4 [file jmir_v25i1e41142_app4.pdf]

Table S3. Baseline characteristics and outcomes of patients with and without cardiac surgery–associated acute kidney injury in the derivation cohort.

Variables	No acute kidney injury (n = 2714)	Acute kidney injury (n = 564)	*P* value
**Demographics**			
Age (year), median (IQR)	1 (0.6-5)	0.5 (0.2-1)	<.001
Sex (male), n (%)	1397 (51.5)	312 (55.3)	.11
Body length (cm), median (IQR)	84 (68-110)	65 (60-78)	<.001
Weight (kg), median (IQR)	10.0 (6.7-17.0)	6.3 (5.0-9.0)	<.001
**ABO blood groups, n (%)**			.33
Type A	914 (33.7)	186 (33)	
Type B	629 (23.2)	115 (20.4)	
Type O	969 (35.7)	213 (37.8)	
Type AB	202 (7.4)	50 (8.9)	
**Preoperative conditions**			
Cyanotic heart disease, n (%)	530 (19.5)	210 (37.2)	<.001
Pulmonary hypertension, n (%)	1456 (53.6)	307 (54.4)	.77
Pulmonary infection, n (%)	222 (8.2)	71 (12.6)	.001
Infective endocarditis, n (%)	34 (1.3)	10 (1.8)	.44
Previous cardiac surgery, n (%)	133 (4.9)	48 (8.5)	.001
Genetic disease, n (%)	57 (2.1)	15 (2.7)	.51
Noncardiac malformation, n (%)	87 (3.2)	24 (4.3)	.26
Preoperative intensive care, n (%)	121 (4.5)	47 (8.3)	<.001
Preoperative length of stay (day), median (IQR)	4 (2-6)	5 (3-8)	<.001
**American Society of Anesthesiologists physical status, n (%)**			<.001
I	20 (0.7)	1 (0.2)	
II	542 (20.1)	47 (8.3)	
III	1639 (60.7)	339 (60.2)	
IV	491 (18.2)	174 (30.9)	
V	10 (0.4)	2 (0.4)	
**Laboratory tests**			
Baseline creatinine (μmol/L), median (IQR)	26.4 (21.3-35.0)	20.6 (17.0-25.6)	<.001
Baseline estimated glomerular filtration rate (mL/min/1.73 m^2), median (IQR)	117.3 (99.7-136.1)	123.3 (99.1-149.6)	<.001
Left ventricular ejection fraction (%), median (IQR)	69 (66-73)	70 (66-74)	.20
Hemoglobin (g/L), median (IQR)	120 (109-129)	113 (102-125)	<.001
Red blood cell distribution width (%), median (IQR)	13.3 (12.7-14.6)	13.9 (13.0-15.3)	<.001
White blood cells ($\times 10^9$/L), median	7.9 (6.4-9.8)	8.4 (6.8-10.3)	<.001

(IQR)			
Platelets (×10^9/L), median (IQR)	312 (252-380)	335 (261-399)	.003
Dipstick albuminuria, n (%)	50 (2.2)	7 (1.5)	.47
Blood urea nitrogen (mmol/L), median (IQR)	4.17 (3.13-5.16)	3.37 (2.34-4.77)	<.001
Total bilirubin (μmol/L), median (IQR)	7.3 (5.1-10.5)	7.9 (5.1-13.9)	<.001
Alanine aminotransferase (U/L), median (IQR)	15.5 (11.3-23.9)	21.2 (14.5-32.5)	<.001
Aspartate aminotransferase (U/L), median (IQR)	33.7 (26.7-43.4)	40.7 (32.2-54.2)	<.001
Albumin (g/L), median (IQR)	40.5 (38.4-42.4)	39.5 (37.3-41.5)	<.001
Potassium (mmol/L), median (IQR)	4.76 (4.43-5.08)	5.00 (4.69-5.38)	<.001
Sodium (mmol/L), median (IQR)	138.5 (137.1-139.8)	137.6 (136.4-138.9)	<.001
Chloride (mmol/L), median (IQR)	103.3 (101.7-104.8)	102.7 (100.9-104.3)	<.001
Calcium (mmol/L), median (IQR)	2.40 (2.31-2.48)	2.44 (2.35-2.53)	<.001
**Preoperative medications, n (%)**			
Iodinated contrast media	309 (11.4)	102 (18.1)	<.001
Digoxin	82 (3)	22 (3.9)	.34
Diuretics	238 (8.8)	78 (13.8)	<.001
Nonsteroidal anti-inflammatory drugs	43 (1.6)	11 (2)	.66
Angiotensin converting enzyme inhibitor/angiotensin II receptor blocker	51 (1.9)	10 (1.8)	1.00
Nephrotoxic antibiotics	50 (1.8)	11 (2)	1.00
Antiviral drugs	135 (5)	28 (5)	1.00
**Intraoperative variables**			
Emergent surgery, n (%)	136 (5)	32 (5.7)	.59
Operation time (min), median (IQR)	150 (125-189)	175 (140-252)	<.001
Perfusion time (min), median (IQR)	55 (43-78)	79 (55-134)	<.001
Cross clamp time (min), median (IQR)	32 (22-47)	44 (30-77)	<.001
Cardioversion, n (%)	245 (9.1)	34 (6)	.024
Lowest mean arterial pressure (mmHg), median (IQR)	35 (31-40)	34 (30-38)	<.001
Lowest core temperature (°C), median (IQR)	33.5 (32.1-34.6)	31.9 (29.9-33.3)	<.001
Intraoperative blood loss (mL/kg), median (IQR)	20.0 (13.8-28.6)	30.8 (22.2-40.0)	<.001
Intraoperative fluid balance (%), median (IQR)	–0.7 (–1.8 to 0.1)	–0.6 (–2.2 to 0.5)	.10
**Risk Adjustment for Congenital Heart Surgery 1 score, n (%)**			<.001

1	458 (17.1)	22 (4)	
2	1688 (63.1)	344 (62.8)	
3	501 (18.7)	152 (27.7)	
4	29 (1.1)	30 (5.5)	
**Outcomes**			
In-hospital mortality, n (%)	13 (0.5)	25 (4.4)	<.001
Intensive care unit length of stay (day), median (IQR)	1 (1-3)	4 (2-9)	<.001
Hospital length of stay (day), median (IQR)	8 (6-11)	12 (8-21)	<.001

Table S4. Baseline characteristics and outcomes of patients with and without cardiac surgery–associated acute kidney injury in the external validation cohort.

Variables	No acute kidney injury (n = 534)	Acute kidney injury (n = 51)	*P* value
**Demographics**			
Age (year), median (IQR)	4 (2-8)	1 (0.5-4.5)	<.001
Sex (male), n (%)	260 (48.7)	28 (54.9)	.48
Body length (cm), median (IQR)	102 (83-130)	72 (63-100)	<.001
Weight (kg), median (IQR)	15.0 (10.0-23.0)	9.0 (5.8-15.0)	<.001
**ABO blood groups, n (%)**			.36
Type A	180 (33.9)	20 (39.2)	
Type B	119 (22.4)	15 (29.4)	
Type O	185 (34.8)	12 (23.5)	
Type AB	47 (8.9)	4 (7.8)	
**Preoperative conditions**			
Cyanotic heart disease, n (%)	90 (16.9)	23 (45.1)	<.001
Pulmonary hypertension, n (%)	217 (40.6)	24 (47.1)	.46
Pulmonary infection, n (%)	18 (3.4)	12 (23.5)	<.001
Infective endocarditis, n (%)	8 (1.5)	1 (2)	1.00
Previous cardiac surgery, n (%)	15 (2.8)	6 (11.8)	.004
Genetic disease, n (%)	10 (1.9)	2 (3.9)	.64
Noncardiac malformation, n (%)	13 (2.4)	2 (3.9)	.86
Preoperative intensive care, n (%)	4 (0.7)	6 (11.8)	<.001
Preoperative length of stay (day), median (IQR)	6 (3-7)	8 (6-10)	<.001
**American Society of Anesthesiologists physical status, n (%)**			<.001
I	0 (0)	0 (0)	
II	118 (22.8)	2 (4.1)	
III	313 (60.4)	26 (53.1)	
IV	87 (16.8)	21 (42.9)	
V	0 (0)	0 (0)	
**Laboratory tests**			
Baseline creatinine (μmol/L), median (IQR)	44.1 (37.0-53.0)	39.0 (34.0-46.0)	.003
Baseline estimated glomerular filtration rate (mL/min/1.73 m^2), median (IQR)	85.5 (73.1-97.1)	73.9 (64.7-95.5)	.015
Left ventricular ejection fraction (%), median (IQR)	66 (62-70)	66 (62-73)	.82
Hemoglobin (g/L), median (IQR)	125 (117-135)	125 (103-173)	.81
Red blood cell distribution width (%), median (IQR)	13.5 (12.9-14.6)	14.5 (13.4-16.4)	.001
White blood cells ($\times 10^9$/L), median	7.5 (6.2-9.1)	9.7 (6.6-11.4)	.001

(IQR)			
Platelets (×10^9/L), median (IQR)	272 (225-325)	270 (211-344)	.73
Dipstick albuminuria, n (%)	4 (0.8)	0 (0)	1.00
Blood urea nitrogen (mmol/L), median (IQR)	4.20 (3.30-5.08)	3.42 (2.48-4.42)	.004
Total bilirubin (μmol/L), median (IQR)	7.1 (5.1-10.7)	8.0 (5.4-14.8)	.14
Alanine aminotransferase (U/L), median (IQR)	14.0 (11.1-18.4)	18.0 (12.8-29.1)	.001
Aspartate aminotransferase (U/L), median (IQR)	31.1 (25.3-38.6)	33.7 (30.1-42.7)	.007
Albumin (g/L), median (IQR)	43.2 (41.2-45.5)	42.2 (38.9-45.2)	.028
Potassium (mmol/L), median (IQR)	4.43 (4.16-4.70)	4.60 (4.36-4.81)	.028
Sodium (mmol/L), median (IQR)	140.0 (138.8-141.2)	139.0 (138.0-140.2)	.005
Chloride (mmol/L), median (IQR)	104.2 (102.8-105.6)	103.5 (102.1-105.1)	.037
Calcium (mmol/L), median (IQR)	2.46 (2.37-2.54)	2.48 (2.36-2.57)	.61
**Preoperative medications, n (%)**			
Iodinated contrast media	61 (11.4)	12 (23.5)	.023
Digoxin	5 (0.9)	6 (11.8)	<.001
Diuretics	28 (5.2)	10 (19.6)	<.001
Nonsteroidal anti-inflammatory drugs	12 (2.2)	0 (0)	.57
Angiotensin converting enzyme inhibitor/angiotensin II receptor blocker	3 (0.6)	1 (2)	.79
Nephrotoxic antibiotics	1 (0.2)	0 (0)	1.00
Antiviral drugs	1 (0.2)	0 (0)	1.00
**Intraoperative variables**			
Emergent surgery, n (%)	10 (1.9)	4 (7.8)	.029
Operation time (min), median (IQR)	190 (160-221)	267 (215-313)	<.001
Perfusion time (min), median (IQR)	61 (45-84)	106 (82-129)	<.001
Cross clamp time (min), median (IQR)	35 (22-52)	58 (41-82)	<.001
Cardioversion, n (%)	65 (12.2)	8 (15.7)	.61
Lowest mean arterial pressure (mmHg), median (IQR)	39 (32-45)	33 (30-40)	.004
Lowest core temperature (°C), median (IQR)	33.4 (32.0-34.8)	31.3 (29.9-33.2)	<.001
Intraoperative blood loss (mL/kg), median (IQR)	19.6 (15.3-25.0)	25.6 (20.1-34.6)	<.001
Intraoperative fluid balance (%), median (IQR)	1.5 (–0.1 to 2.8)	2.8 (–1.0 to 4.8)	.06
**Risk Adjustment for Congenital Heart Surgery 1 score, n (%)**			.05

1	91 (17.4)	1 (2.1)	
2	349 (66.9)	37 (78.7)	
3	76 (14.6)	8 (17)	
4	6 (1.1)	1 (2.1)	
**Outcomes**			
In-hospital mortality, n (%)	1 (0.2)	4 (7.8)	<.001
Intensive care unit length of stay (day), median (IQR)	1 (1-2)	6 (3-11)	<.001
Hospital length of stay (day), median (IQR)	8 (7-9)	12 (9-17)	<.001
