# Supplementary material for: Machine Learning–Based Prediction of Acute Kidney Injury Following Pediatric Cardiac Surgery: Model Development and Validation Study
Source: J Med Internet Res. 2023 Jan 5;25:e41142. doi: 10.2196/41142 (PMC9893730; doi:10.2196/41142)
Supplement: Multimedia Appendix 5 [file jmir_v25i1e41142_app5.pdf]

Table S5. Preoperative variables selected by the 4 feature selection methods.

Variables	Least Absolute Shrinkage and Selection Operator	Boruta algorithm	Recursive feature elimination	Filtering
Age	√	√	√	√
Body length	√	√	√	√
Weight		√	√	√
Cyanotic heart disease	√	√	√	√
Pulmonary infection	√	√	√	√
Previous cardiac surgery	√	√	√	√
Preoperative intensive care		√	√	√
Preoperative length of stay	√	√	√	√
American Society of Anesthesiologists physical status	√	√	√	√
Baseline creatinine	√	√	√	√
Baseline estimated glomerular filtration rate	√	√	√	√
Hemoglobin	√	√	√	√
Red blood cell distribution width		√	√	√
Platelets	√	√	√	√
Blood urea nitrogen		√	√	√
Total bilirubin	√	√	√	√
Alanine aminotransferase		√	√	√
Aspartate aminotransferase	√	√	√	√
Albumin	√		√	√
Potassium	√	√	√	√
Sodium		√	√	√
Chloride	√	√	√	√
Calcium	√	√	√	√
Iodinated contrast media	√	√	√	√
Diuretics		√	√	√

Table S6. Preoperative and intraoperative variables selected by the 4 feature selection methods.

Variables	Least Absolute Shrinkage and Selection Operator	Boruta algorithm	Recursive feature elimination	Filtering
Age		√	√	√
Body length	√	√	√	√
Weight		√	√	√
Cyanotic heart disease	√	√	√	√
Previous cardiac surgery		√	√	√
Preoperative length of stay	√	√	√	√
American Society of Anesthesiologists physical status	√		√	√
Baseline creatinine	√	√	√	√
Baseline estimated glomerular filtration rate	√	√	√	√
Hemoglobin	√	√	√	√
Platelets	√	√	√	√
Total bilirubin	√	√	√	√
Alanine aminotransferase		√	√	√
Aspartate aminotransferase	√	√	√	√
Albumin	√		√	√
Potassium	√	√	√	√
Sodium		√	√	√
Chloride		√	√	√
Calcium	√	√	√	√
Diuretics		√	√	√
Operation time	√	√	√	√
Perfusion time	√	√	√	√
Cross clamp time		√	√	√
Lowest mean arterial pressure		√	√	√
Lowest core temperature		√	√	√
Intraoperative blood loss	√	√	√	√
Risk Adjustment for Congenital Heart Surgery 1 score	√	√	√	√
