# Supplementary material for: Machine Learning–Based Prediction of Acute Kidney Injury Following Pediatric Cardiac Surgery: Model Development and Validation Study
Source: J Med Internet Res. 2023 Jan 5;25:e41142. doi: 10.2196/41142 (PMC9893730; doi:10.2196/41142)
Supplement: Multimedia Appendix 6 [file jmir_v25i1e41142_app6.pdf]

Table S7. Performance of the machine learning models in cross-validation.

Models, models	Area under the receiver operating characteristic curve, mean (SD)
**Models with only the preoperative variables**	
K-nearest neighbor	0.756 (0.017)
Naive bayes	0.739 (0.019)
Support vector machines	0.736 (0.021)
Random forest	0.786 (0.022)
Extreme gradient boosting	0.795 (0.021)
Neural networks	0.787 (0.020)
**Models with the preoperative and intraoperative variables**	
K-nearest neighbor	0.811 (0.018)
Naive bayes	0.792 (0.019)
Support vector machines	0.786 (0.025)
Random forest	0.825 (0.017)
Extreme gradient boosting	0.832 (0.018)
Neural networks	0.827 (0.017)
