# Supplementary material for: Machine Learning–Based Prediction of Acute Kidney Injury Following Pediatric Cardiac Surgery: Model Development and Validation Study
Source: J Med Internet Res. 2023 Jan 5;25:e41142. doi: 10.2196/41142 (PMC9893730; doi:10.2196/41142)

Figure S2. Precision-recall curves of the extreme gradient boosting models for cardiac surgery–associated acute kidney injury. (A) Precision-recall curve of the extreme gradient boosting model with only the preoperative variables. (B) Precision-recall curve of the extreme gradient boosting model with the preoperative and intraoperative variables. AUC, area under the curve.

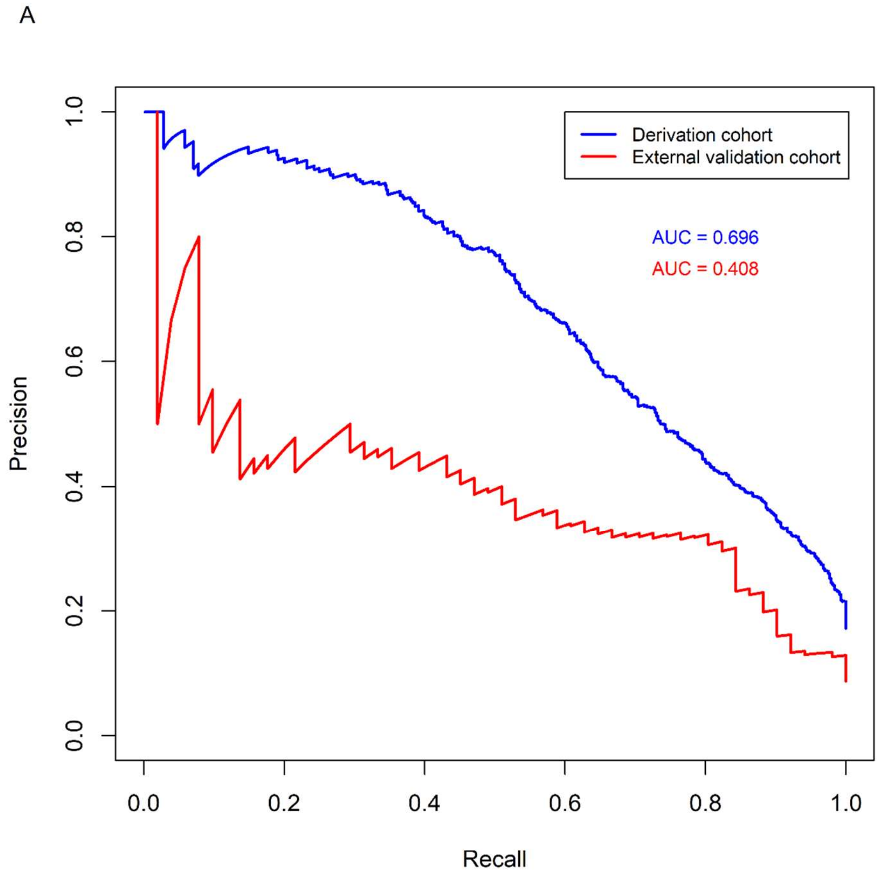

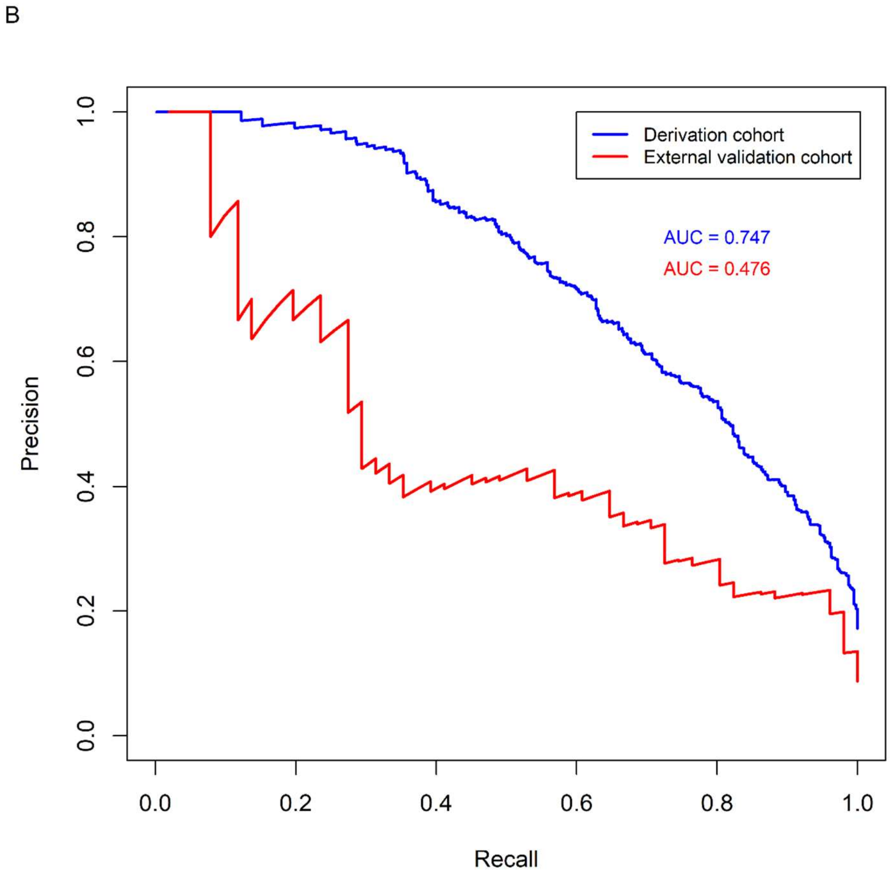

Figure S3. Calibration curves of the extreme gradient boosting models for cardiac surgery–associated acute kidney injury. (A) Calibration curve of the extreme gradient boosting model with only the preoperative variables. (B) Calibration curve of the extreme gradient boosting model with the preoperative and intraoperative variables.

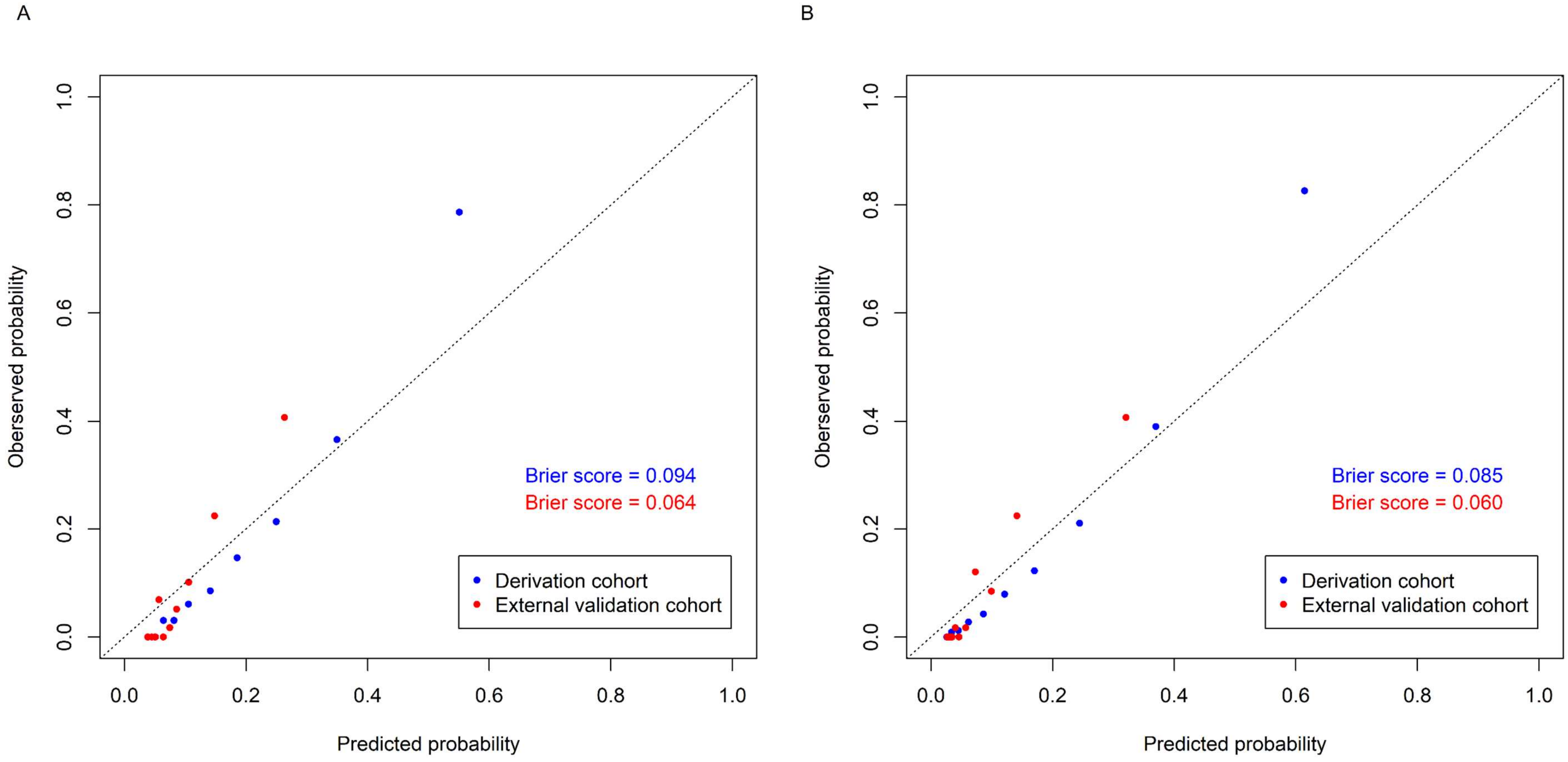

Supplement: Multimedia Appendix 7 [file jmir_v25i1e41142_app7.pdf]
