# Supplementary material for: Machine Learning–Based Prediction of Acute Kidney Injury Following Pediatric Cardiac Surgery: Model Development and Validation Study
Source: J Med Internet Res. 2023 Jan 5;25:e41142. doi: 10.2196/41142 (PMC9893730; doi:10.2196/41142)

Figure S4. Shapley additive explanations dependence plots for the association between the predictors and cardiac surgery–associated acute kidney injury in the extreme gradient boosting model with the combined data set. eGFR: estimated glomerular filtration rate; SHAP: Shapley additive explanations.

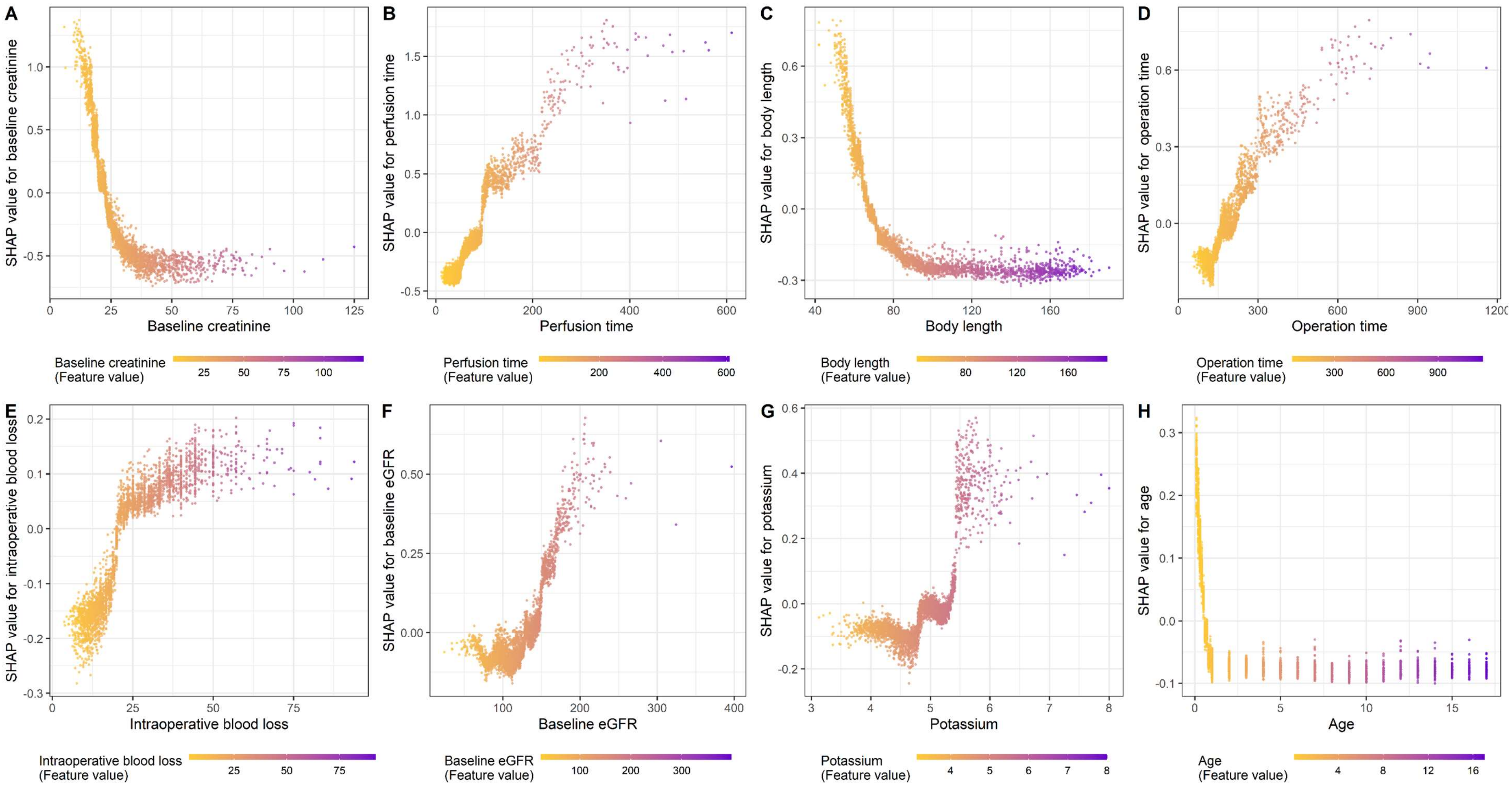

Supplement: Multimedia Appendix 8 [file jmir_v25i1e41142_app8.pdf]
