# Supplementary material for: Machine Learning–Based Prediction of Acute Kidney Injury Following Pediatric Cardiac Surgery: Model Development and Validation Study
Source: J Med Internet Res. 2023 Jan 5;25:e41142. doi: 10.2196/41142 (PMC9893730; doi:10.2196/41142)

Figure S5. Receiver operating characteristic curves of the extreme gradient boosting models for cardiac surgery–associated acute kidney injury stages 2-3. (A) Receiver operating characteristic curve of the extreme gradient boosting model with only the preoperative variables. (B) Receiver operating characteristic curve of the extreme gradient boosting model with the preoperative and intraoperative variables. AUC, area under the curve.

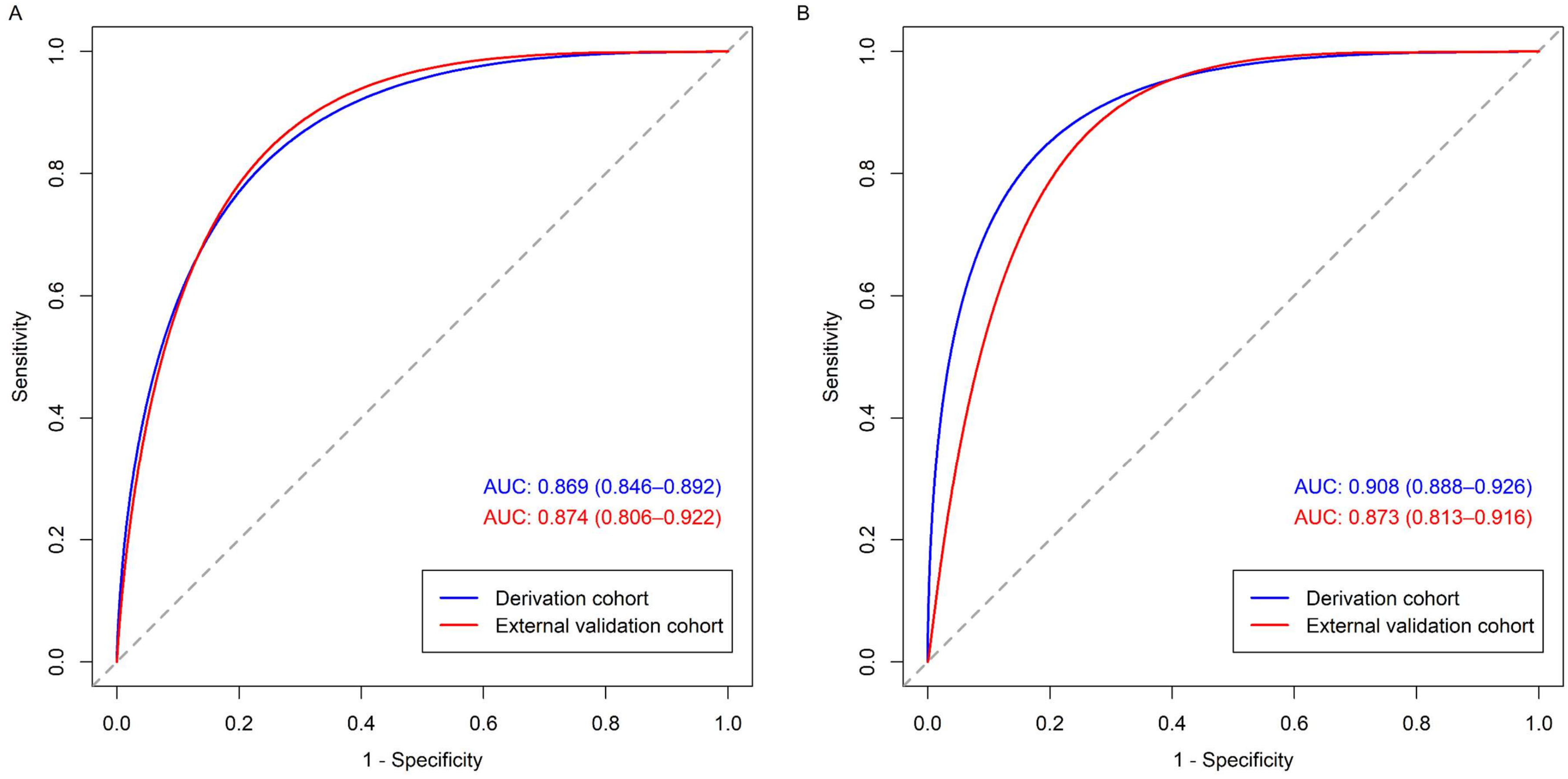

Supplement: Multimedia Appendix 9 [file jmir_v25i1e41142_app9.pdf]
