# Supplementary material for: Machine Learning–Based Prediction of Acute Kidney Injury Following Pediatric Cardiac Surgery: Model Development and Validation Study
Source: J Med Internet Res. 2023 Jan 5;25:e41142. doi: 10.2196/41142 (PMC9893730; doi:10.2196/41142)

Figure S6. Receiver operating characteristic curves of the extreme gradient boosting models for cardiac surgery–associated acute kidney injury in patients in infancy. (A) Receiver operating characteristic curve of the extreme gradient boosting model with only the preoperative variables. (B) Receiver operating characteristic curve of the extreme gradient boosting model with the preoperative and intraoperative variables. AUC, area under the curve.

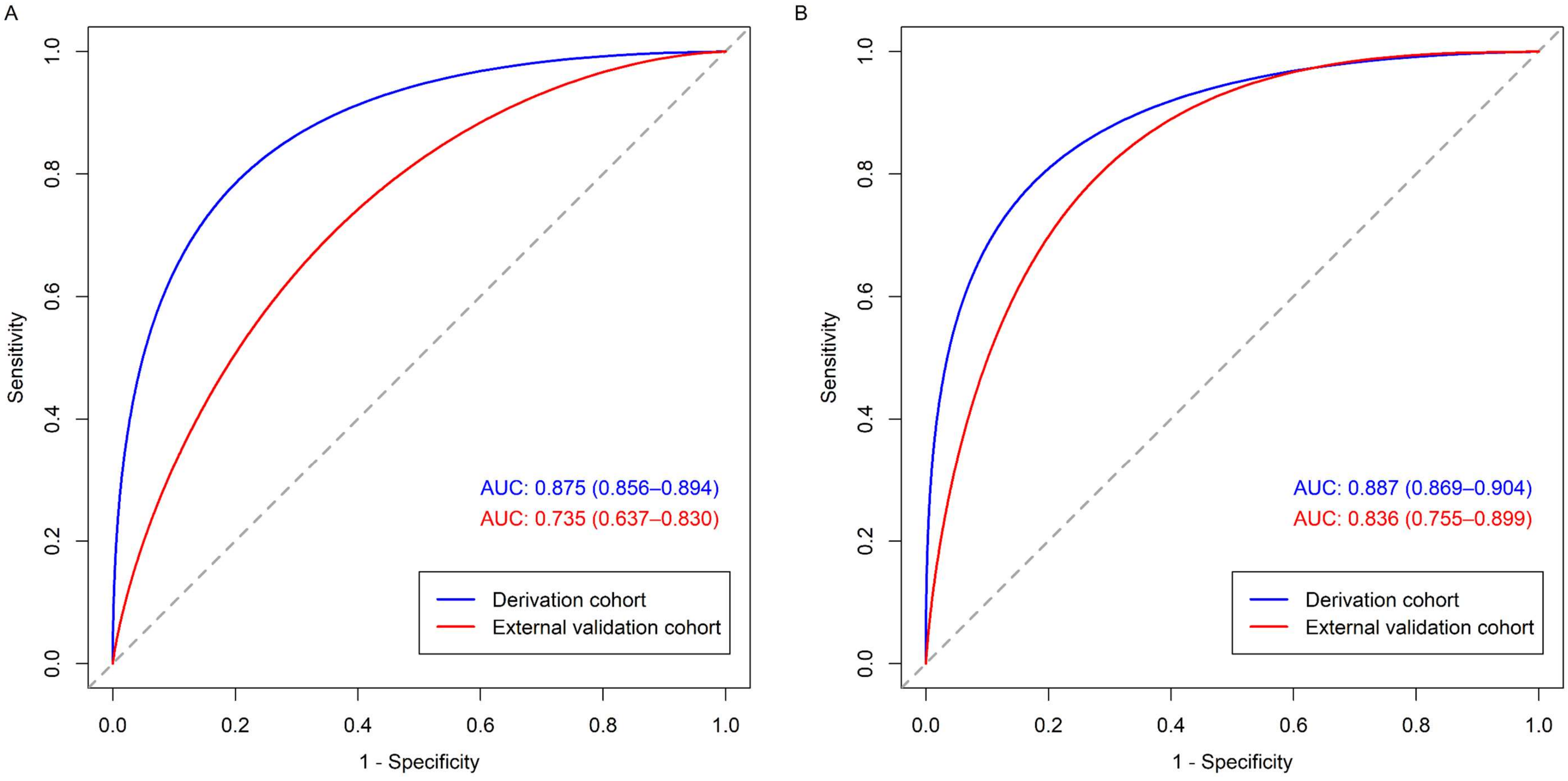

Figure S7. Receiver operating characteristic curves of the extreme gradient boosting models for cardiac surgery–associated acute kidney injury in patients in childhood. (A) Receiver operating characteristic curve of the extreme gradient boosting model with only the preoperative variables. (B) Receiver operating characteristic curve of the extreme gradient boosting model with the preoperative and intraoperative variables. AUC, area under the curve.

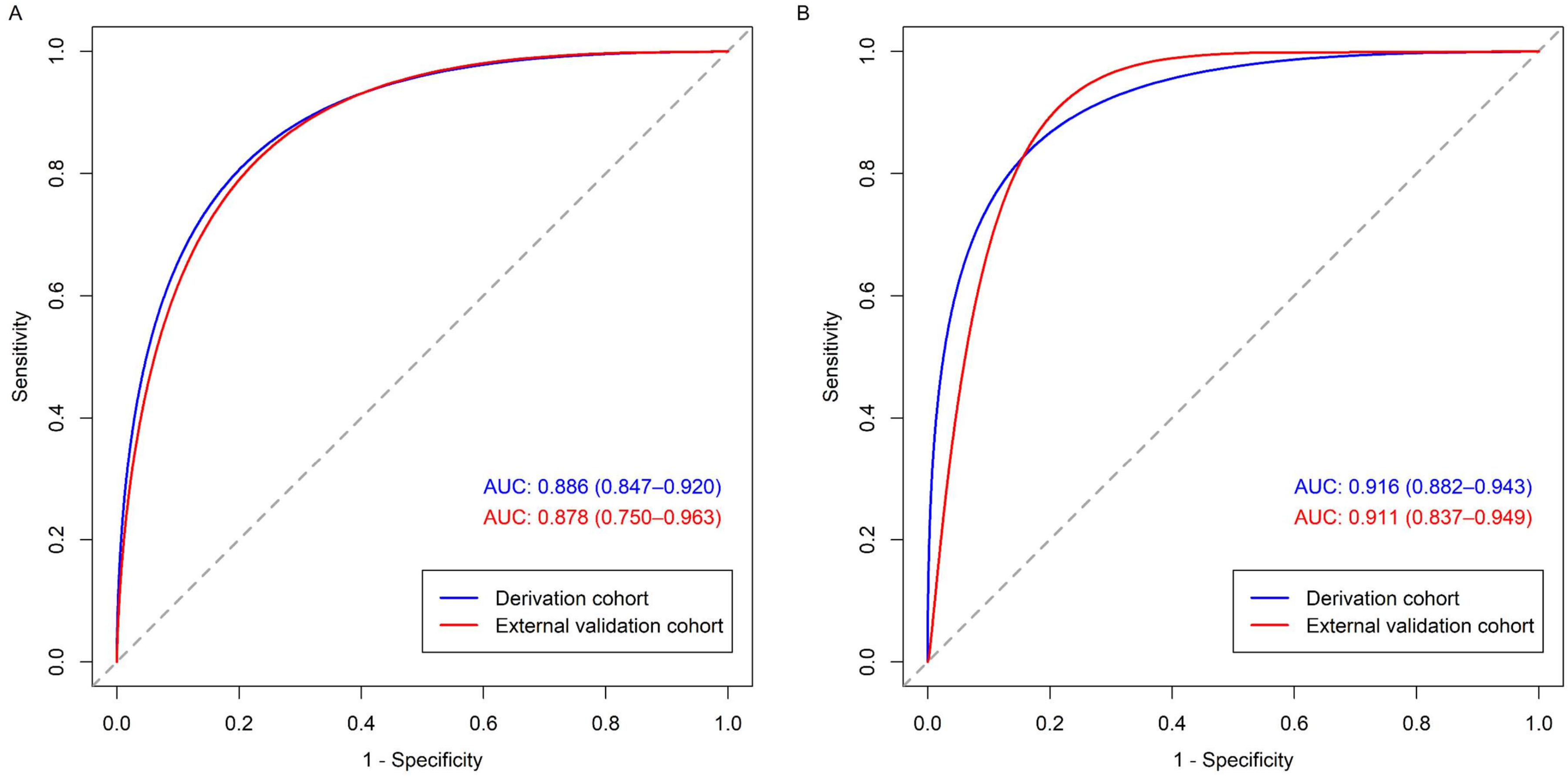

Figure S8. Receiver operating characteristic curves of the extreme gradient boosting models for cardiac surgery–associated acute kidney injury in patients in adolescence. (A) Receiver operating characteristic curve of the extreme gradient boosting model with only the preoperative variables. (B) Receiver operating characteristic curve of the extreme gradient boosting model with the preoperative and intraoperative variables. AUC, area under the curve.

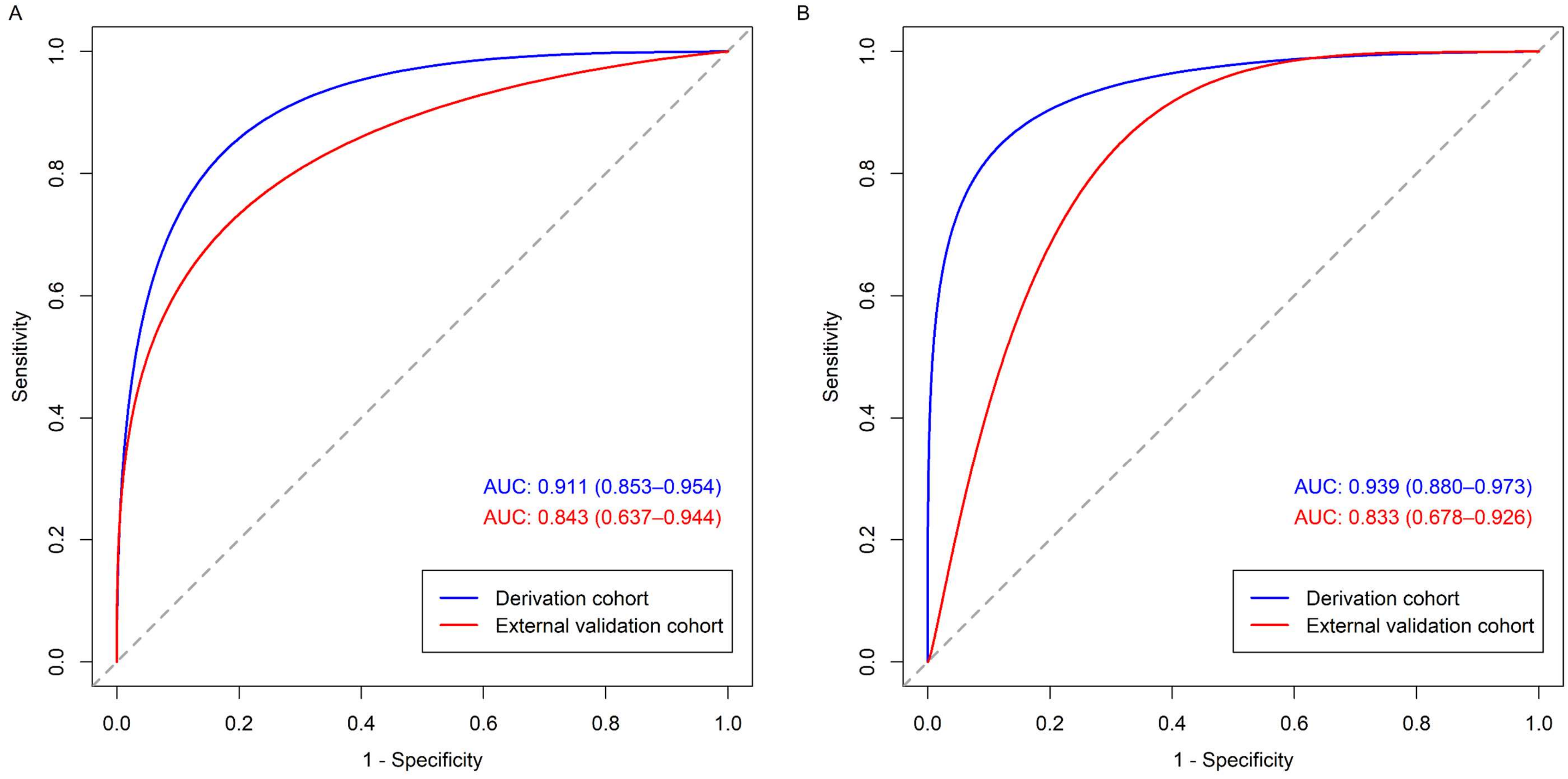

Figure S9. Receiver operating characteristic curves of the extreme gradient boosting models for cardiac surgery–associated acute kidney injury in patients with a Risk Adjustment for Congenital Heart Surgery 1 score of 2 or lower. (A) Receiver operating characteristic curve of the extreme gradient boosting model with only the preoperative variables. (B) Receiver operating characteristic curve of the extreme gradient boosting model with the preoperative and intraoperative variables. AUC, area under the curve.

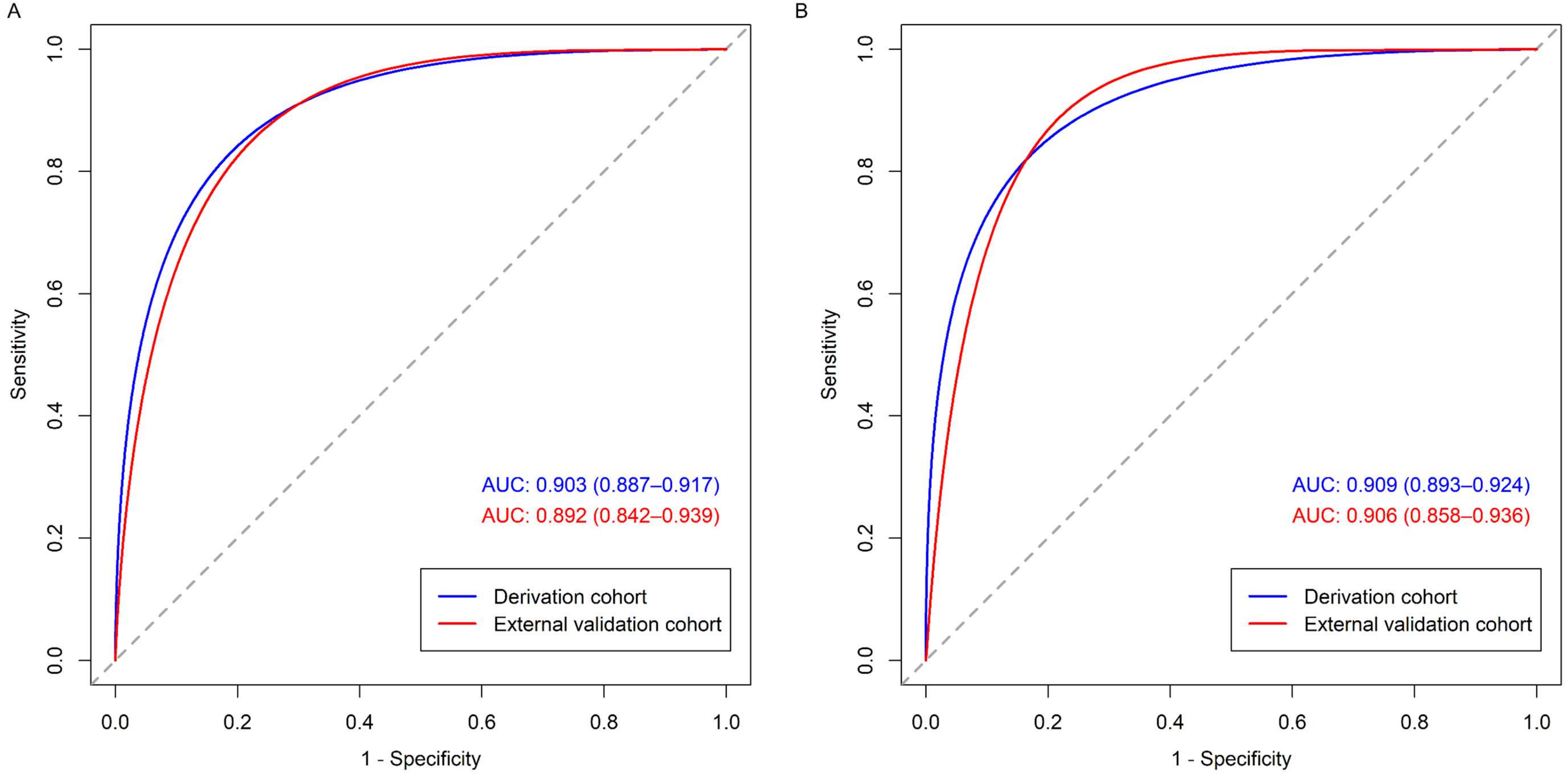

Figure S10. Receiver operating characteristic curves of the extreme gradient boosting models for cardiac surgery–associated acute kidney injury in patients with a Risk Adjustment for Congenital Heart Surgery 1 score of 3 or higher. (A) Receiver operating characteristic curve of the extreme gradient boosting model with only the preoperative variables. (B) Receiver operating characteristic curve of the extreme gradient boosting model with the preoperative and intraoperative variables. AUC, area under the curve.

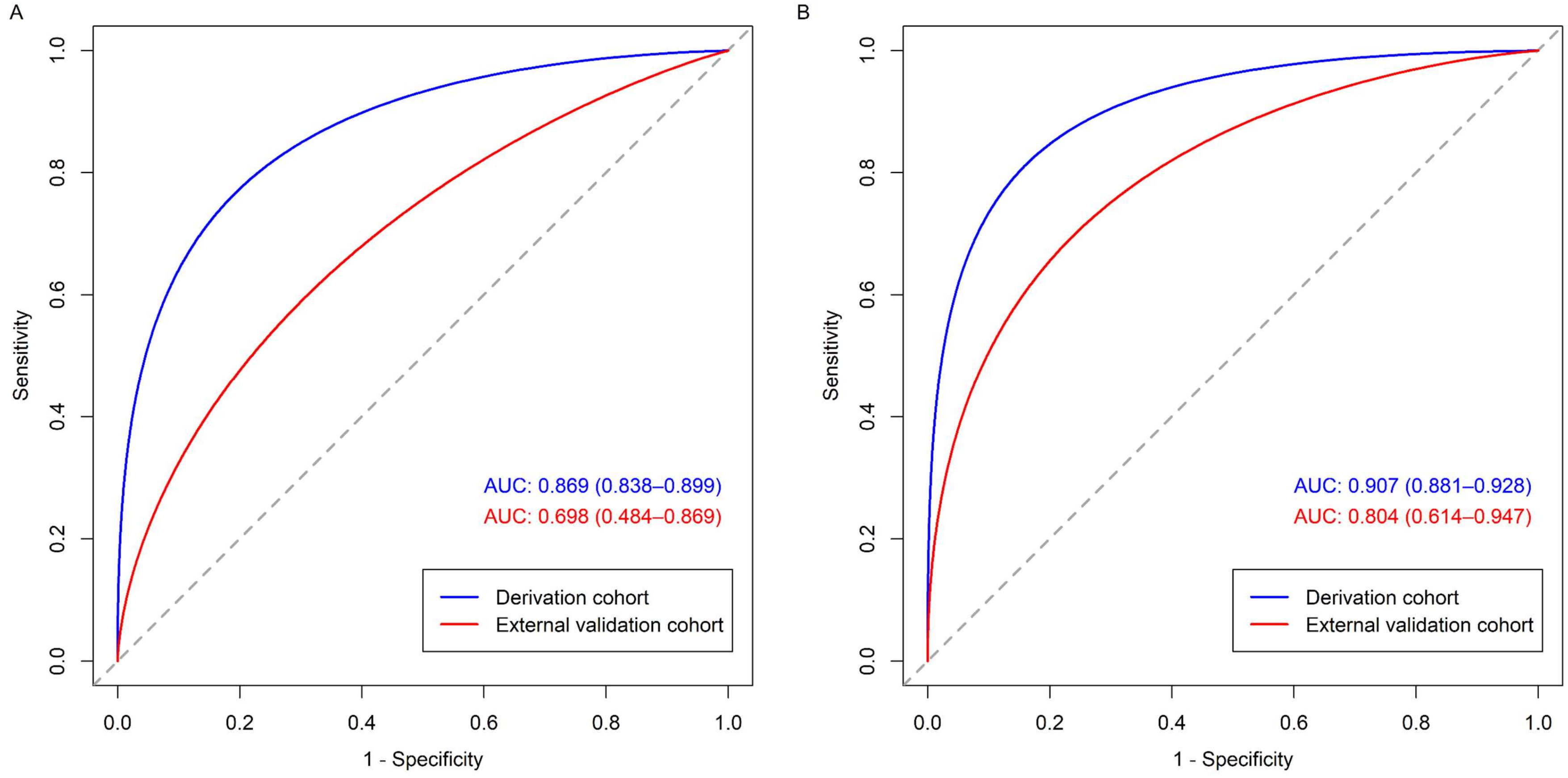

Supplement: Multimedia Appendix 10 [file jmir_v25i1e41142_app10.pdf]
