# Supplementary material for: Machine Learning–Based Prediction of Acute Kidney Injury Following Pediatric Cardiac Surgery: Model Development and Validation Study
Source: J Med Internet Res. 2023 Jan 5;25:e41142. doi: 10.2196/41142 (PMC9893730; doi:10.2196/41142)

Figure S11. Receiver operating characteristic curves of the extreme gradient boosting models for cardiac surgery–associated acute kidney injury trained on the balanced derivation cohort by up-sampling. (A) Receiver operating characteristic curves of the extreme gradient boosting model with only the preoperative variables. (B) Receiver operating characteristic curves of the extreme gradient boosting model with the preoperative and intraoperative variables. AUC, area under the curve.

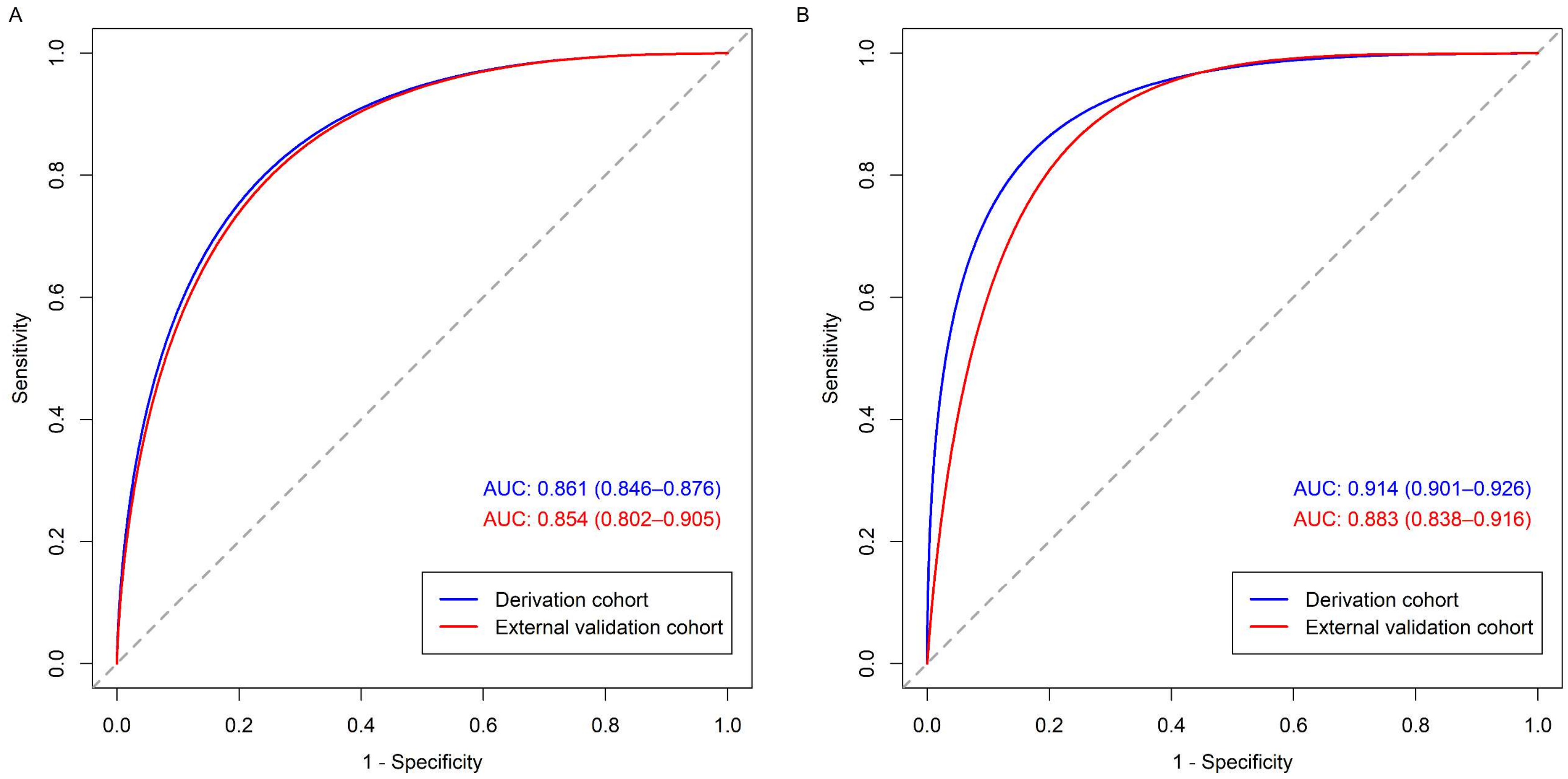

Figure S12. Receiver operating characteristic curves of the extreme gradient boosting models for cardiac surgery–associated acute kidney injury trained on the balanced derivation cohort by down-sampling. (A) Receiver operating characteristic curves of the extreme gradient boosting model with only the preoperative variables. (B) Receiver operating characteristic curves of the extreme gradient boosting model with the preoperative and intraoperative variables. AUC, area under the curve.

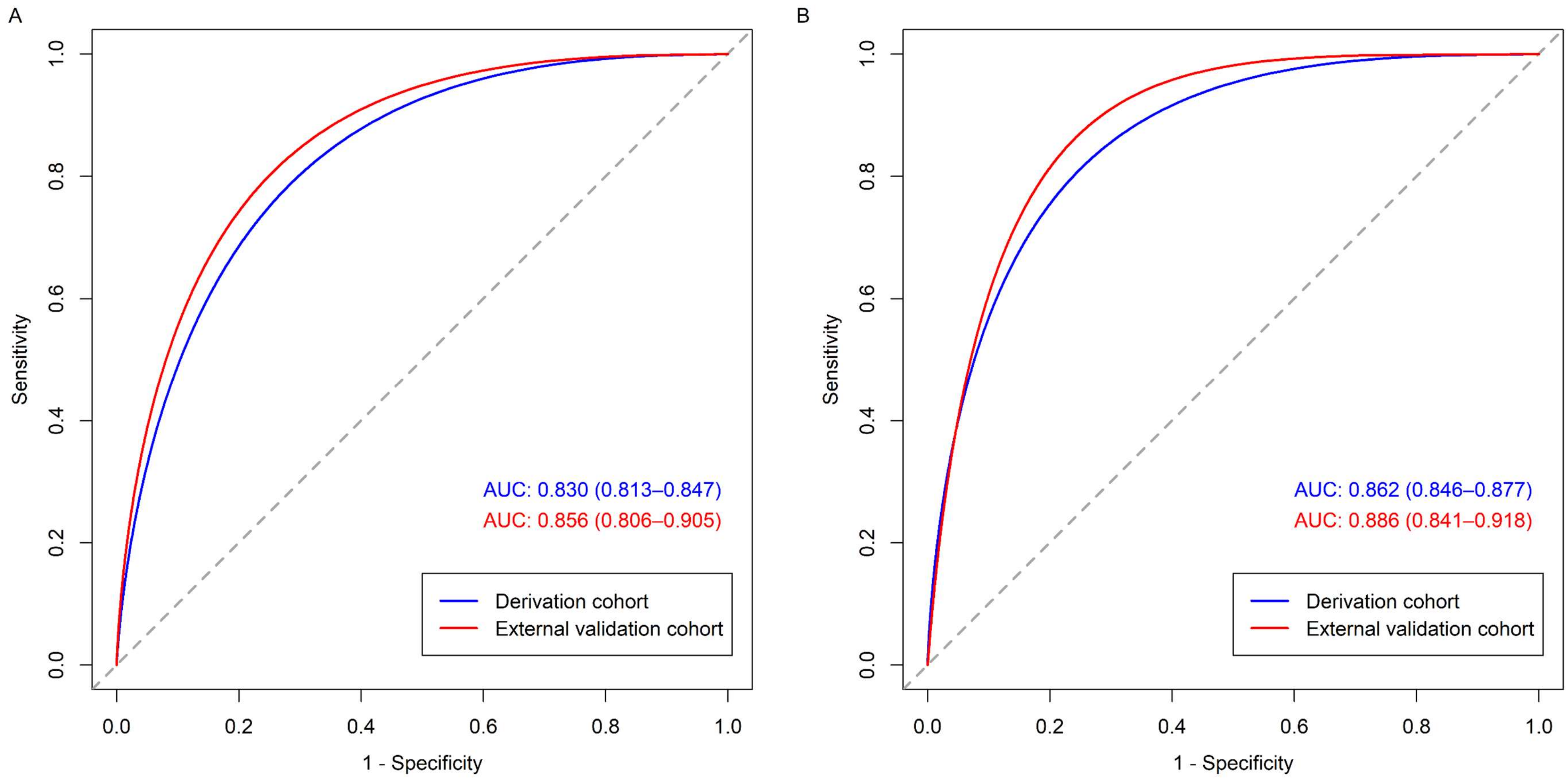

Supplement: Multimedia Appendix 11 [file jmir_v25i1e41142_app11.pdf]
